# Patient and Caregiver Reported Barriers to Cancer Clinical Trial Participation: A Scoping Review

**DOI:** 10.1002/cnr2.70700

**Published:** 2026-10-13

**Authors:** Sarah Scruton, Chisato Ito, Julia Bohnen, Robin Urquhart, Chiquita Hessels, Summer Konechny, Rosilene Kraft, Randy Thompson, Cheryl Ho, Deirdre Weymann, Yvonne Bombard, Emanuel Krebs, Avram Denburg, Timothy P. Hanna, Howard J. Lim, Saadia Shareen, Dean A. Regier, Samantha Pollard

**Affiliations:** ^1^ Cancer Outcomes Research Program Nova Scotia Health Halifax Nova Scotia Canada; ^2^ Faculty of Health Sciences Simon Fraser University Vancouver British Columbia Canada; ^3^ BC Children's Hospital Research Institute Vancouver British Columbia Canada; ^4^ Department of Community Health and Epidemiology Dalhousie University Halifax Nova Scotia Canada; ^5^ Li Fraumeni Syndrome Association Canada Canada; ^6^ pan‐Canadian organization Canada; ^7^ Department of Medical Oncology BC Cancer Vancouver British Columbia Canada; ^8^ Population Health Sciences BC Cancer Research Institute Vancouver British Columbia Canada; ^9^ Genomics Health Services Research Program Unity Health Toronto Toronto Ontario Canada; ^10^ Institute of Health Policy, Management and Evaluation University of Toronto Toronto Ontario Canada; ^11^ Division of Hematology/Oncology The Hospital for Sick Children Toronto Ontario Canada; ^12^ Division of Cancer Care and Epidemiology Sinclair Cancer Research Institute Kingston Ontario Canada; ^13^ Department of Oncology Queen's University Kingston Ontario Canada; ^14^ School of Population and Public Health University of British Columbia Vancouver British Columbia Canada

**Keywords:** barriers, clinical trials, informed consent, oncology, participant preferences, precision medicine, trial design

## Abstract

**Background:**

The development of cancer treatments relies on patient participation in clinical trials, yet poor recruitment and retention hinder evidence generation. There exists an urgent need for trial designs that address patient‐identified barriers, informational gaps, and concerns. The goal of this evidence synthesis was to identify patient‐ and caregiver‐reported barriers to trial participation and retention.

**Methods:**

We conducted a search in PubMed, Ovid MEDLINE, CINAHL Complete, Scopus, and Web of Science to identify studies describing patient‐ and caregiver‐reported barriers to cancer clinical trial participation. Eligible studies included qualitative or quantitative research published in the English language that reported barriers experienced by individuals with cancer or their caregivers. Four authors screened titles and abstracts, followed by full‐text review and data abstraction of included studies. Qualitative results were summarized narratively, and quantitative findings were described using descriptive statistics. Barriers and recommendations for improved clinical trial design were categorized into thematic groups.

**Results:**

The search yielded 7918 studies, with 131 meeting inclusion criteria. Barriers to trial participation were grouped into three overarching themes: (1) psychosocial barriers (e.g., fear of adverse events, distrust, and perceived limited personal benefit); (2) logistical barriers (e.g., travel requirements and financial burden); and (3) informational barriers (e.g., challenges understanding consent materials). Recommendations emphasized financial and logistical support for participants, appropriate information provision, and fostering trust between patients and trial staff.

**Conclusion:**

Addressing informational, psychosocial, and logistical obstacles to participation is essential for timely evidence generation. Without meaningful strategies to engage patients and caregivers in clinical trial designs, evidence generation for novel oncology innovations will continue to lag, hampering patient access and appropriate reimbursement.

## 1 Introduction

Alongside improvements in cancer prevention, diagnostics, and treatment, survival across multiple cancer types has improved dramatically over the past 30 years [1, 2, 3, 4, 5, 6, 7]. These advancements have been driven by numerous innovations, many of which have emerged through clinical trials generating evidence on safety, effectiveness, and value of novel interventions [8, 9, 10]. Precision oncology trials, seeking to match patients to treatments based on their individual tumor characteristics identified through genetic and genomic sequencing, have further contributed to improvements in cancer care [11, 12]. Precision oncology increasingly offers the potential for unprecedented improvements in cancer outcomes by expanding treatment options for select patient populations, such as those with rare cancers, specific tumor sub‐types, and actionable molecular alterations [13, 14, 15, 16, 17, 18]. Despite promise, access to precision oncology innovations such as targeted therapeutics and comprehensive genomic profiling remains mostly accessible within research settings, with broader integration into clinical practice lagging behind [19, 20].

Access to emerging therapies relies on the generation of evidence from clinical trials to support regulatory approval and reimbursement decisions. National and international regulatory bodies review results from such trials to assess potential risks of new therapies and provide regulatory approval based on evidence of safety, efficacy, and quality [8, 9, 10]. Drug agencies then review evidence for effectiveness, cost‐effectiveness, patient value, and feasibility of adoption to generate reimbursement recommendations [21, 22, 23].

While evidence from individual level patient data is a necessary prerequisite to regulatory and reimbursement decision making, clinical trials face challenges in recruiting and retaining patient participants. For example, the Canadian Partnership Against Cancer reports that adult clinical trial participation among newly diagnosed patients ranges from just 1% to 5.8% [24]. Reasons for limited participation in cancer clinical trials are not fully understood, but recent investigations have identified that costs, inconveniences, and inflexibility of trial protocols (e.g., visit schedules and travel requirements) may bear on patient willingness and abilities to participate [25, 26, 27]. This breadth of barriers aligns with ongoing challenges to enroll and retain patients in oncology clinical trials, including lengthy accrual periods, participant loss to follow up, and withdrawals [2, 28, 29, 30, 31]. Collectively, such challenges hamper robust and timely evidence generation. As such, identifying and addressing trial participation barriers is critical to designing mitigation strategies for future protocols [32, 33, 34, 35, 36, 37, 38, 39, 40, 41, 42].

In an effort to improve clinical trial design in light of patient informational, process, and logistical needs and preferences, the perspectives of those asked to participate are required. This includes the perspectives of both patients and their informal caregivers, who play a critical yet seldom formally acknowledged role in clinical trial participation and retention [43]. Missing from the current evidence base is a comprehensive understanding of patient and caregiver reported barriers to trial participation. Without a clear understanding of these barriers, person‐centered improvements to clinical trial design to strengthen recruitment and retention cannot be effectively achieved.

The objective of this scoping review was to identify barriers to participation in therapeutic clinical trials in oncology, from the perspectives of patients with cancer and informal caregivers, to inform the design of more accessible and patient‐centered trials. As a secondary objective, we examined motivators for participation and recommendations for improving trial access.

## 2 Methods

### 2.1 Eligibility

#### 2.1.1 Inclusion Criteria

This scoping review included studies reporting patient‐ or caregiver‐reported barriers to participation in cancer treatment clinical trials. Eligible studies included primary research using any study design in which individuals diagnosed with cancer or caregivers were asked to select, rate, describe, or otherwise report barriers to trial participation (e.g., through interviews, focus groups, or surveys). Caregivers were defined as individuals providing unpaid physical, emotional, financial, or other support to someone with cancer (e.g., a partner, family member, or friend) [44]. Studies of subgroups outside the target population (e.g., healthcare providers) were included if findings for eligible participants were reported separately.

Barriers were defined as factors explicitly described by participants as impeding or restricting trial access, negatively influencing the decision to participate, or creating burden during the trial experience. Studies were eligible regardless of whether participants reported previous trial experience and no age restrictions were applied; therefore, pediatric, adolescent, and adult populations were included. For pediatric studies, barriers were extracted based on the reported perspectives of patients or caregivers. Consideration was given to assent, parental permission, and family decision‐making when these were explicitly reported as barriers to trial participation.

Studies from all countries and healthcare settings were eligible, as limiting inclusion to Canadian studies would have restricted the evidence due to the limited volume of Canadian research in this area. Country and region were extracted to characterize the geographic distribution of the literature; however, differences in healthcare systems were not a primary focus of this review. Only peer‐reviewed literature published in English with full text available and accessible to the research team was included.

#### 2.1.2 Exclusion Criteria

Studies were excluded if barriers to clinical trial participation were not directly elicited or reported by patients or caregivers. Studies examining the associations between demographic, clinical, or socioeconomic factors and trial participation or non‐participation were excluded unless these characteristics were explicitly described as barriers by participants.

Cancer treatment trials were the focus of this review because participation decisions in therapeutic trials involve unique considerations related to treatment access, risk–benefit evaluation, and clinical decision making that may differ from non‐treatment research. Therefore, studies focused on non‐treatment cancer trials, including supportive care interventions (e.g., exercise programs, chemoprevention, or complementary/alternative medicines), were excluded. Studies focused solely on the development or validation of interventions, measurement tools, decision aids, recruitment strategies, or other implementation resources were excluded, along with survey development or instrument validation studies.

Review articles were excluded; however, their included primary studies were screened for eligibility. Theses/dissertations, conference abstracts without full peer‐reviewed publications, preprints, and other grey literature were excluded.

### 2.2 Search Strategy

The search strategy was developed based on a preliminary search of the literature. One analyst (Scruton) performed a limited search of Ovid MEDLINE to identify 15 key papers on the topic. Relevant keywords, index terms, and MeSH headings were extracted to inform the final search string. Given the broad indexing of these studies and the use of a single database, the search strategy was developed to be comprehensive and highly sensitive. One author (Ito) conducted the final search of five databases (Ovid MEDLINE, PubMed, Scopus, CINAHL Complete, and Web of Science) using the developed search strategy (see Supplementary Appendix 1). The search was not limited to a specific date range and is current to June 12, 2026. A review protocol was developed by the research team to guide the conduct of the scoping review; however, it was not prospectively registered or published.

### 2.3 Study Selection

A single reviewer uploaded all identified studies into Covidence and removed duplicates [45]. Following the JBI methodology for scoping reviews [46], study selection began with title and abstract screening, followed by full‐text review of potentially eligible studies. The research coordinator (Scruton) and principal investigator (Pollard) first pilot‐tested the eligibility criteria by independently screening a subsample of abstracts (10%) until substantial agreement was achieved (Cohen's kappa = 0.8). The research coordinator then repeated this calibration process with two additional reviewers (Ito and Bohnen), independently screening 10% of the abstracts until substantial agreement was achieved. Following calibration, the remaining abstracts were divided among the four reviewers and screened independently according to the predefined eligibility criteria. This approach was selected to improve the feasibility and timeliness of screening while maintaining consistency through reviewer training and calibration [47].

Full texts of potentially eligible articles were then retrieved for review. Again, the four reviewers first assessed a sub‐sample of full texts until consensus was reached (Cohen's kappa = 0.8). Remaining full texts were then divided and reviewed in full, with disagreements resolved through discussion. Reasons for exclusion of studies that did not meet the inclusion criteria were recorded. Finally, one reviewer (Scruton) screened reference lists of included studies to capture relevant papers not captured by the formal search. A PRISMA flow diagram illustrating the study selection process is reported in Figure 1.

**FIGURE 1:**
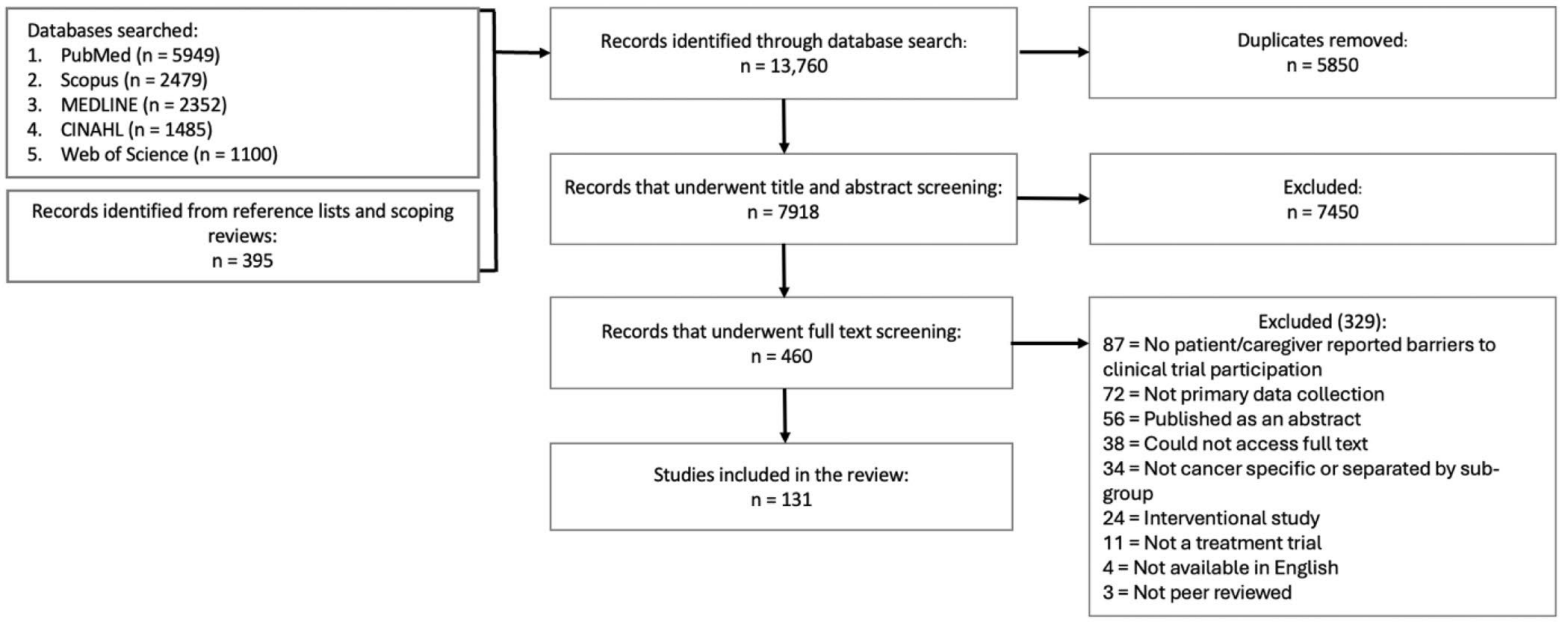
PRISMA diagram.

### 2.4 Data Extraction and Synthesis

A standardized data extraction form was developed by the research team. Four reviewers (Scruton, Bohnen, Ito, and Shareen) first pilot‐tested the form by independently extracting data from approximately 10%–15% of included studies until consensus was reached. Following calibration, the remaining studies were divided among the four reviewers for independent data extraction to improve feasibility and timeliness. Extracted data included publication date, location, study population, sample size, study setting, design, trial type, and patient‐ and caregiver‐reported barriers to clinical trial participation. If reported, we also abstracted data on participant‐reported facilitators and recommendations to improve clinical trial design or participation. Where possible, studies reporting results quantitatively were summarized using descriptive statistics, while qualitative study results were reported according to the method presented (e.g., thematic or content analysis). Two authors (Scruton and Pollard) identified themes representing groups of related barriers to trial participation, through an iterative process of independent review and discussion. Related motivators for participation were reported in a heat map and patient‐reported recommendations were summarized descriptively. Of note, our reporting of participant characteristics and results aligns with terminology presented in each individual study (e.g., African American vs. Black, trial decliners vs. trial refusers). Results are reported following PRISMA guidelines for scoping reviews (PRISMA‐ScR) [48]. Consistent with JBI methodology for scoping reviews [46], a critical appraisal of included studies was not undertaken. The objective of this review was to map and characterize the existing evidence on barriers to trial participation, rather than to evaluate the methodological quality of individual studies [46, 47, 49, 50].

## 3 Results

The search yielded 7918 individual studies. A total of 131 studies published between 1979 and 2026 met the inclusion criteria, with 50% published between 2010 and 2019 (see Table 1) [25, 26, 27, 38, 40, 51, 52, 53, 54, 55, 56, 57, 58, 59, 60, 61, 62, 63, 64, 65, 66, 67, 68, 69, 70, 71, 72, 73, 74, 75, 76, 77, 78, 79, 80, 81, 82, 83, 84, 85, 86, 87, 88, 89, 90, 91, 92, 93, 94, 95, 96, 97, 98, 99, 100, 101, 102, 103, 104, 105, 106, 107, 108, 109, 110, 111, 112, 113, 114, 115, 116, 117, 118, 119, 120, 121, 122, 123, 124, 125, 126, 127, 128, 129, 130, 131, 132, 133, 134, 135, 136, 137, 138, 139, 140, 141, 142, 143, 144, 145, 146, 147, 148, 149, 150, 151, 152, 153, 154, 155, 156, 157, 158, 159, 160, 161, 162, 163, 164, 165, 166, 167, 168, 169, 170, 171, 172, 173, 174, 175, 176, 177]. Approximately half (49.6%; *n* = 65) of the studies were conducted in the United States. A total of 13 studies (9.9%) focused on early‐phase trials, while the vast majority (83.2%; *n* = 109) did not specify the trial phase. Similarly, 114 studies (87.0%) did not specify the trial intervention type. Slightly less than half (45.0%; *n* = 59) of the studies employed quantitative cross‐sectional designs, while the remainder used qualitative or mixed methods. Sample sizes ranged from 3–66 953 participants. Most studies focused solely on individuals with a cancer diagnosis (90.1%; *n* = 118), while 13 studies (9.9%) included caregivers. In 73 studies (55.7%), most participants had not participated in clinical trials. Regarding cancer type, 67 studies (51.1%) included multiple cancer types, 19 (14.5%) focused on breast cancer, and 24 (18.3%) did not report cancer type. Most studies included exclusively (19.8%; *n* = 26) or mostly female participants (45.8%; *n* = 60), and one study included only male participants. A total of 53 studies (40.5%) included a majority of participants reporting White ethnicity and 56 (42.7%) did not report race/ethnicity. A smaller number of studies included mostly African American/Black participants (8.4%; *n* = 11) or participants of other race/ethnicity (8.4%; *n* = 11). A detailed description of study characteristics is provided in Table 2.

We identified three themes related to barriers to clinical trial participation: (1) psychosocial barriers, (2) logistical barriers, and (3) informational barriers, with embedded sub‐themes illustrated in Figures 2, 3, 4. Additional themes are summarized in Supplementary Appendix 2, describing minor themes that were identified from a minority of studies.

**TABLE 1:** Study characteristics and participant demographics.

Characteristic		*n*	%
Country	United States	65	49.6
	UK	17	13.0
	Canada	7	5.3
	Australia	6	4.6
	China	6	4.6
	Denmark	5	3.8
	South Korea	4	3.1
	Germany	2	1.5
	Ireland	2	1.5
	Poland	2	1.5
	Saudi Arabia	2	1.5
	Other^†^	13	9.9
Trial phase	Early phase (I/I‐II)	13	9.9
	Multiple phases	5	3.8
	Phase III	4	3.1
	Not specified	109	83.2
Cancer treatment type	Chemotherapy	4	3.1
	Multimodal	3	2.3
	Radiotherapy	2	1.5
	Immunotherapy	1	0.8
	Stem cell transplantation	1	0.8
	Surgery	1	0.8
	Not specified	114	87.0
	Various	5	3.8
Study design	Quantitative	59	45.0
	Qualitative	54	41.2
	Mixed methods	18	13.7
Sample size	1–99	69	52.7
	100–499	42	32.1
	1000+	13	9.9
	500–999	7	5.3
Caregivers included in sampling frame	No	118	90.1
	Yes	13	9.9
Current or past clinical trial participation	> 50% no (declined or not offered)	73	55.7
	> 50% yes (accepted or taken part)	23	17.6
	50/50 yes and no	2	1.5
	Not reported	33	25.2
Cancer type	Breast	19	14.5
	Ovary	4	3.1
	Colorectal	2	1.5
	Lymphoma	2	1.5
	Prostate	2	1.5
	Bladder	1	0.8
	Bone	1	0.8
	Ductal carcinoma in situ	1	0.8
	Endometrial	1	0.8
	Esophageal	1	0.8
	Laryngeal and pharyngeal	1	0.8
	Leukemia	1	0.8
	Liver	1	0.8
	Lung	1	0.8
	Mouth	1	0.8
	Solid tumor	1	0.8
	Various	67	51.1
	Not reported	24	18.3
Sex/gender	> 50% women/female	60	45.8
	> 50% men/male	30	22.9
	100% women/female	26	19.8
	50/50 female/male	5	3.8
	100% men/male	1	0.8
	Not reported	9	6.9
Race/ethnicity	Majority White	53	40.5
	Majority African American/Black	11	8.4
	Majority other race/ethnicity	11	8.4
	Not reported	56	42.7

*Note:* ^†^Includes Brazil, Bulgaria, France, Greece, India, Italy, Japan, Lebanon, Mexico, Portugal, Puerto Rico, Singapore, Slovakia, Slovenia, Spain, Sweden, Syria, Netherlands, and Turkey.

**TABLE 2:** Detailed study characteristics and participant demographics.

Author, year	Country	Trial phase	Intervention type	Study design	Sample size	Cancer type	Mean age (range)^a^	Sex/gender^b^	Race/ethnicity	Trial experience^c^	Caregiver included
Abhyankar 2016 [51]	UK	Phase III	Not specified	Qualitative	21	Breast: 52.4% Ovarian: 38.1% Endometrial 9.5%	57 (29–81)	Female: 100%	Not reported	Not reported	No
Abrahão 2022 [52]	United States	Not specified	Not specified	Qualitative	26	Leukemia: 46% Lymphoma: 16% Sarcoma: 18% Female breast: 8% Gastrointestinal: 12%	31 (18–42)	Female: 53% Male: 46%	Non‐Hispanic White: 84% Hispanic: 8% Asian: 8%	Yes: 100%	No
Adams 2025 [53]	United States	Not specified	Not specified	Qualitative	4	Ductal carcinoma in situ	58.3 (54–65)	Female: 100%	White: 75.0% Black: 0.0% Asian: 0.0% Hispanic: 25.0%	No: 100%	No
Akkad 2026 [54]	United States	Not specified	Not specified	Cross‐sectional	301	Lymphoma	61.6 (SD: 13.9)	Female: 42.9% Male: 57.1%	Non‐Hispanic White: 34% African American: 32% Hispanic: 33%	Yes: 37% No: 63%	No
Alhajji 2022 [55]	United States	Not specified	Various	Cross‐sectional	41	Not reported	60.5	Female: 51% Male: 49%	African American/Black: 100%	Yes: 39.0% No: 61.0% Declined: 14.0%	No
Almutairi 2017 [56]	Saudi Arabia	Not specified	Not specified	Cross‐sectional	244	Colorectal: 21.7% Breast: 28.3% Lung: 8.6% Prostate: 5.7% Skin: 0.4% Lymphoma: 7.4% Leukemia: 3.7% Ovarian: 6.6% Head/neck: 3.3% Other: 14.3%	50.83 (SD: 14.1)	Female: 57% Male: 43%	Not reported	Not reported	No
Alotaibi 2025 [57]	Saudi Arabia	Not specified	Not specified	Cross‐sectional	201	Breast: 32.3% Lymphoma: 17.9% Colorectal: 7.5% Renal: 3.0% Prostate: 2.5% Lung: 2.5% Other: 34.3%	29.5 (14–84)	Female: 69.2% Male: 30.8%	Not reported	Not reported	No
Anderson 2020 [58]	United States	Not specified	Not specified	Cross‐sectional	1264	Breast: 41% Lymphoma: 10% Gynecologic: 11% Thyroid: 23% Melanoma: 14%	32.6 (15–40)	Female: 100%	Non‐Hispanic White: 74% Non‐Hispanic Black: 6% Hispanic: 13% Other: 7%	Yes: 5% No: 95%	No
Andrews 2022 [59]	United States	Not specified	Not specified	Qualitative	40	Breast	Patients: 57 (33–79) Advocates: 61 (36–76)	Female: 100%	Patients: Black/African American: 41.7% White: 58.3% Advocates: Black/African American: 12.5% White: 87.5%	Not reported	No
Arana‐Chicas 2025 [60]	United States	Not specified	Not specified	Qualitative	27	Breast: 8.7% Gastrointestinal: 56.6% Genitourinary: 8.7% Gynecologic: 4.3% Lung: 21.7%	73.4	Female: 59.3% Male: 40.7%	Non‐Hispanic White: 96.3% Hispanic: 3.7%	Not reported	No
Avis 2006 [61]	United States	Not specified	Not specified	Cross‐sectional	183	Breast	51.5 (30–84)	Female: 100%	Non‐Hispanic White: 75.8% Black: 7.7% Hispanic: 14.3% Asian American: 2.2%	Yes: 57.9% Declined: 42.1%	No
Ayodele 2016 [62]	Ireland	Not specified	Not specified	Cross‐sectional	219	Breast: 30% < 65 years; 13% > 65 years GI: 35% < 65 years; 47% > 65 years Lung: 14% < 65 years; 24% > 65 years Genitourinary: 5% < 65 years; 4% > 65 years Others: 5% < 65 years; 3% > 65 years NA: 2% < 65 years; 2% > 65 years	(31–82)	Female: 62.1% Male: 37.9%	Not reported	Yes: 11.9% No: 84.0% Declined: 8.7%	No
Barakat 2014 [63]	United States	Phase III	Not specified	Qualitative	29	Rhabdomyosarcoma: 15.4% Osteosarcoma: 38.5% Acute lymphoblastic leukemia: 23.1% Acute promyelocytic leukemia: 15.4% Non‐Hodgkin lymphoma: 7.6%	17.54 (15–24)	Female: 53.8% Male: 46.2%	Ethnicity: Not Hispanic or Latino: 11 (84.6%) Hispanic or Latino: 2 (15.4%) Race: White: 10 (76.9%) Black or African American: 1 (7.7%) Other: 2 (15.4%)	Yes: 69.2%	No
Barofky 1979 [64]	United States	Not specified	Multimodal	Qualitative	76	Extremity soft‐tissue sarcoma: 34.0% Head–neck‐trunk soft‐tissue sarcoma: 28.2% Primary osteosarcoma: 37.9%	Majority < = 19	Female: 35.0% Male: 65.0%	White: 84.5% Black: 15.5%	Declined: 14.0%	No
Bartoszkiewicz 2021 [65]	Poland	Not specified	Not specified	Cross‐sectional	100	Breast	<40: 50% >40: 50%	Female: 100%	Not reported	Yes: 9.0% No: 91.0%	No
Basche 2008 [25]	United States	Early phase (I/I‐II)	Not specified	Cross‐sectional	300	Not reported	Majority 65–74	Female: 51.5% Male: 48.5%	White: 93.7% African American: 3.3% Hispanic or Latino: 2.0% Other: 1.0%	Not reported	No
Bell 2018 [66]	Canada	Multiple phases	Not specified	Qualitative	21	Testes: 14% Leukemia: 14% Lymphoma: 29% Breast: 14% Sarcoma: 29%	Median: 31 (18–39)	Female: 52% Male: 48%	Not reported	Yes: 48.0% No: 43.0%	No
Brehaut 2021 [67]	Canada	Not specified	Not specified	Cross‐sectional	210	Breast	57.0 (31–84)	Female: (97.5%) Male: 0.8% Missing: 1.6%	White/Caucasian: 91.4% Asian: 1.2% South Asian: 1.2% Black: 0.8% Arab/West Asian: 0% First Nations/Indigenous: 1.6% Filipino: 0% Latin American: 0.4% Other (e.g., participant reported): 2.0% Prefer not to answer: 1.2%	Yes: 18.0% No: 56.1% Declined: 54.0%	No
Brown 2013 [68]	United States	Multiple phases	Not specified	Mixed methods	22	Breast: 45.5% Colon/rectum/stomach: 22.7% Mouth/throat: 13.6% Other (ovary, lung, bone marrow): 18.1%	57 (39–77)	Female: 68.2% Male: 31.8%	African American/Black: 100%	No: 100% Declined: 100%	No
Byrne 2014 [26]	United States	Not specified	Not specified	Cross‐sectional	1100	SD: 11.7 Breast: 42.8% Prostate: 28.7% Colorectal: 17.9% Lung: 10.6%	67.4	Female: 57.3% Male: 42.7%	Hispanic: 38.1% White: 35.1% Black: 26.8%	Yes: 7.7% No: 92.3%	No
Catania 2008 [40]	Italy	Not specified	Not specified	Cross‐sectional	102	Breast: 62.7% Lung: 37.2%	Median: 58 (21–78)	Female: 73.5% Male: 26.5%	Not reported	No: 100%	No
Catt 2011 [69]	UK	Early phase (I/I‐II)	Not specified	Qualitative	40	Colorectal/upper GI: 55% Breast: 15% Gynecological: 13% Skin: 8% Other: 10%	58.8 (29–76)	Female: 45% Male: 55%	Not reported	Yes: 90% Declined: 10%	No
Chalela 2014 [70]	United States	Early phase (I/I‐II)	Not specified	Cross‐sectional	100	Not reported	55.19 (SD: 12.7)	Female: 51% Male: 49%	Non‐Hispanic White: 47% Hispanic: 19% Other: 14%	Yes: 50%	No
Chen 2025 [71]	United States	Not specified	Not specified	Cross‐sectional	1150	Breast	53.9 (SD: 11.9)	Not reported	Asian: 4.4% Black: 19.5% Hispanic: 3.1% White: 73.0%	Yes: 64.3% No: 35.7%	No
Cohen 2007 [27]	United States	Early phase (I/I‐II)	Not specified	Mixed methods	Survey: 100 Interview: 16	Not reported	Interview: 57 (29–79) Survey: 57 (20–80)	Interview: Female: 38% Male: 62%, Survey: Female: 52% Male: 48%	Interviews: White: 88% Other: (12%) Survey: White: 86% Other: 14%	Not reported	No
Cox 1996 [72]	UK	Early phase (I/I‐II)	Not specified	Qualitative	7	Not reported	(48–74)	Female: 28.6% Male: 71.4%	Not reported	Yes: 100%	No
Cox 1999 [73]	UK	Early phase (I/I‐II)	Not specified	Mixed methods	55	Gastrointestinal tract: 29% Breast: 27% Lung: 24% Ovary: 16% Others: 4%	Majority < 59	Female: 60% Male: 40%	Not reported	Yes: 100%	No
Cox 2000 [74]	UK	Early phase (I/I‐II)	Not specified	Mixed methods	55	Majority lung, breast, gastrointestinal	58.8 (37–74)	Female: 60% Male: 40%	Not reported	Yes: 100%	No
Coyne 2004 [75]	United States	Not specified	Not specified	Qualitative	17	Breast: 29.4% Gynecological: 35.3% Leukemia: 11.8% Colorectal: 5.9% Llung: 5.9% Liver: 5.9% Lymphoma: 5.9%	Majority 50–60+	Female: 88.2% Male: 11.8%	White: 100%	No: 100%	No
Czornik 2024 [76]	Germany	Not specified	Surgery	Qualitative	11	Esophageal	60.2 (32–82)	Female: 18% Male: 82%	Not reported	Not reported	No
Dias 2016 [77]	United States	Not specified	Not specified	Cross‐sectional	85	Lung: 41.6% Melanoma: 22.52% Breast: 15.7% Colon/Rectum: 6.7% Pancreas/others: 13.5%	Median: 60 (29–84)	Female: 48.3% Male: 51.7%	White: 81.82% African American: 18.18%	Not reported	No
Dolinsky 2006 [78]	United States	Not specified	Not specified	Cross‐sectional	157	Breast	Enrolled: 50.9 Never enrolled: 51.8	Enrolled: Fema: 100% Never enrolled: Female: 98.3%	Caucasian: 87% African American: 6% Asian: 2.5% Hispanic: 2% Unknown: 2%	Yes: 73.9% No: 26.1%	No
Fairley 2024 [79]	United States	Not specified	Not specified	Mixed methods	Survey: 257 Focus group: 48	Breast	Focus group: Majority 25–34 Survey: Majority 35–44	Focus group: Female: 100% Survey: Female: 99.6% Male: 0.4%	Black/African American: 100%	Yes: Focus group: 0% Survey: 37% No: Focus groups: 100% Surveys: 63%	No
Fleury 2021 [80]	United States	Not specified	Not specified	Cross‐sectional	933	Not reported	Not reported	Female: 73.1% Male: 26.9%	White: 92.8% Black: 3.6% Asian/Pacific Islander: 2.1% Native American: 1.5%	Yes: 12.4%	No
Gansler 2012 [81]	United States	Not specified	Various	Cross‐sectional	1987	Breast: 11.1% Head and neck: 4.2% Thoracic: 12.9% Melanoma: 5.7% Gastrointestinal: 23.8% Bone and soft tissue: 4.1% Gynecologic: 7.4% Genitourinary 14.7% Intracranial and nervous system: 4.1% Lymphoma: 3.4% Leukemia: 3.1% Myeloma: 1.2% Skin (non‐melanoma): 0.9% Unknown: 1.5% Other: 1.8%	Majority 60–79	Female: 49.3% Male: 50.7%	American Indian or Alaska Native: 0.8% Asian, pacific Islander, or Native Hawaiian: 2.7% Black/African American: 9.2% Hispanic/Latino: 5.0% Non‐Hispanic White: 80.7% Other or mixed race: 1.7%	Yes: 11.0% No: 89.0%	No
Garcea 2005 [82]	UK	Not specified	Chemotherapy	Cross‐sectional	63	Colorectal	Mean ranged from 58.5 to 67.5 across subgroups	Not reported	Not reported	Yes: 36.5% Declined: 63.5%	No
Geana 2017 [83]	United States	Not specified	Not specified	Qualitative	66	Not reported	Inner‐city: 54.77 (28–69) Rural: 67.15 (37–89)	Female: 62.1% Male: 37.9%	White: 71.2% African American: 25.8% Hispanic: 3.0%	Yes: 9.1% No: 90.9%	No
Go 2006 [84]	United States	Not specified	Not specified	Cross‐sectional	1012	Not reported	67 (20–99)	Female: 52.0% Male: 48.0%	White: 99.7% Black: 0.3%	Yes: 4.2% No: 65.8% Declined: 21.7%	No
Granda‐Cameron 2022 [85]	United States	Not specified	Not specified	Qualitative	13	Not reported	Majority 61–80	Female: 92.3% Male: 7.7%	African American/Black: 100%	Not reported	No
Harrison 2007 [86]	Australia	Not specified	Multimodal	Cross‐sectional	103	Colorectal	65	Women: 44% Men: 56%	Not reported	Not reported	No
Haynes‐Maslow 2014 [87]	United States	Not specified	Not specified	Qualitative	82	Breast: 59% Colon: 13% Ovarian: 8% Cervical: 5% Lung: 5% Skin: 5% Lymphoma: 10% Leg: 3% NR: 3%	57 (21–85)	Female: 100%	African American/Black: 100%	Yes: 13.0% No: 87.0%	Yes
Hernandez 2022 [88]	United States	Not specified	Not specified	Qualitative	25	Bone: 4.0% Breast: 68.0% Lung: 8.0% Prostate: 4.0% Nasopharyngeal carcinoma: 4.0% Colon: 12.0%	56.78 (SD: 11.18)	Female: 78.3% Male: 21.7%	African American/Black: 100%	Not reported	No
Hillyer 2020 [89]	United States	Not specified	Not specified	Cross‐sectional	150	Not reported	61.5 (27–96)	Female: 59.3% Male: 40.7%	White: 61.3% Mixed race: 16.7% African American/Black: 13.3% Asian/Pacific Islander: 5.3% Other: 4.0% Hispanic/Latino: 29.3%	Yes: 14.0% No: 85.0%	No
Hillyer 2021 [90]	United States	Not specified	Not specified	Cross‐sectional	67	Not reported	Majority 65	Female: 4.5% Male: 95.5%	Black: 41.8% Other: 58.2%	Not reported	No
Hoadley 2024 [91]	United States	Not specified	Not specified	Cross‐sectional	244	Not reported	60.89 (SD: 10.24)	Female: 63.1% Male: 36.9%	American Indian or Alaskan Native: 0.4% Asian: 0.8% Black/African American: 38.9% White: 55.7% Mixed: 4.1% Hispanic/Latino: 4.7%	No: 100%	No
Ingersgaard 2018 [92]	Denmark	Not specified	Chemotherapy	Qualitative	16	Leukemia	Patients: (14–19) Parents: Not reported	Patients: Male: 100% Parents: Female: 72.7% Male: 27.3%	Not reported	Yes: 80.0% No: 20.0%	Yes
Javid 2012 [93]	United States	Not specified	Not specified	Cross‐sectional	311	Breast	Majority < 65	Female: 100%	Not reported	Yes: 13.2% No: 66.3% Declined: 20.1%	No
Jenkins 2000 [94]	UK	Not specified	Various	Cross‐sectional	204	Breast: 55% Prostate: 11% Testicular: 7% Lung: 3% Colorectal: 7% Ovarian: 9% Melanoma: 1% Lymphoma: 1% Bladder: 2% Pancreas: 2.5% Brain: 0.5%	Majority 45–64	Female: 69.6% Male: 30.4%	Not reported	Yes: 5.4%	No
Jiang 2019 [95]	China	Not specified	Various	Cross‐sectional	199	Lung: 43.7% Breast: 17.6% Lymphoma: 17.6% Gastrointestinal: 6.0%	Majority 40–59	Female: 54.8% Male: 45.2%	Not reported	Yes: 100%	No
Johnston 2025 [96]	UK	Early phase (I/I‐II)	Not specified	Qualitative	101	Leukemia:5.9% Lymphoma: 26.7% Brain or spinal cord tumor: 6.9% Bone or soft tissue sarcoma: 9.9% Germ cell tumor: 5.0% Carcinoma: 24.8% Other/not sure: 20.8%	Adolescent and young adult: Majority 19–24 Older adult: Majority 30+	Adolescent and young adult: Female: 64% Male: 36% Older adult: Female: 43% Male: 57%	Adolescent and young adult: Asian: 2% White: 96% Other: 2% Older adult: Asian: 2% White: 96% Other: 2%	Yes: Adolescent and young adult: 20% Older adult: 65% No: Adolescent and young adult: 72% Older adult: 35%	No
Kearns 2020 [97]	Ireland	Not specified	Not specified	Cross‐sectional	1089	Not reported	59 (18–89)	Female: 64% Male: 35%	Irish: 94% Irish traveler: < 1% Other European: 4% Asian: 1% African: < 1% Other: < 1%	Yes: 25.0% No: 75.0% Declined: 1.0%	No
Kessel 2018 [98]	Germany	Not specified	Not specified	Cross‐sectional	771	Bone/spine cancer/metastases: 4.5% Brain tumors/metastases: 19.7% Breast cancer and gynecological tumors: 7.7% Head and neck cancer: 28.0% Hematological cancer: 9.9% Hepato‐pancreato‐biliary cancer: 6.2% Prostate cancer and urological tumors: 3.9% Skin cancer: 5.7% Upper and lower gastrointestinal cancer: 7.5% Other: 3.0% Unknown: 3.9%	Median: 61 (18–91)	Female: 50.5% Male: 49.3% Unknown: 0.2%	Not reported	Yes: 17.8% No: 80.8% Declined: 12.7%	No
Keusch 2014 [99]	United States	Not specified	Stem cell transplantation	Qualitative	23	Not reported	Patients: 56 (18–70) Parents: 43.5 (28–54)	Patients: Female: 23.5% Male: 76% Parents: Female: 66.8% Male: 33.3%	Not reported	Yes: 100%	Yes
Kim 2008 [100]	South Korea	Not specified	Not specified	Cross‐sectional	524	Breast: 32.1% Lung: 18.7% Stomach: 9.0% Colorectal: 13.4% Hepatocellular: 2.7% Biliary: 4.0% Pancreas: 2.7% Lymphoma: 2.3% Head and neck: 4.4% Sarcoma: 2.1% Gynecology and genitourinary: 2.1% esophagus: 1.5% Others: 5.2%	Majority 60 to < 70	Female: 54.4% Male: 45.6%	Not reported	Not reported	No
Kogan 2022 [101]	Canada	Not specified	Not specified	Qualitative	19	Not reported	58.5 (41–77)	Women: 57.9% Men: 42.1%	Not reported	Yes: 5.3%	No
Kohara 2010 [102]	Japan	Early phase (I/I‐II)	Not specified	Mixed methods	25	Colon: 24% Lung: 20% Breast: 16% Head and neck: 8% Renal: 8% Esophagus: 4% Pancreas: 4% Biliary tract: 4% Ovary: 4% Liposarcoma: 4% Thymoma: 4%	Median: 60 (32–75)	Female: 44% Male: 56%	Japanese: 100%	Yes: 84.0% Declined: 16.0%	No
Kong 2017 [103]	China	Not specified	Not specified	Cross‐sectional	331	Lymphoma	Agreed: 46.9 (SD: 1.18) Disagreed: 43.8 (SD: 1.31)	Female: 37.5% Male: 62.5%	Chinese: 100%	Yes: 53.5% Declined: 46.5%	No
Kouli 2026 [104]	Syria	Not specified	Not specified	Mixed methods	322	Gynecological: 33.5% Breast: 19.9% Urological: 15.2% Other: 31.4%	52.9 (SD: 12.5)	Female: 56.2% Male: 43.8%	Not reported	Not reported	No
Kubilay Tolunay 2025 [105]	Turkey	Not specified	Not specified	Cross‐sectional	1360	Gastrointestinal: 31.3% Breast: 24.6% Lung: 17.7% Genitourinary: 17.1% Head and neck: 2.2% Lymphoma: 1.8% Other malignant neoplasms: 3.5% Missing:1.8%	Median: 52 (18–82)	Female: 49.0% Male: 49.6% Missing: 1.4%	Not reported	Yes: 17.8% No: 82.0%	No
Kumar 2022 [106]	United States	Not specified	Not specified	Cross‐sectional	147	Not reported	Not reported	Female: 86% Male: 14%	White: 35.4% Black: 64.6%	Yes: 17.0% No: 83.0%	No
Lee 2012 [107]	South Korea	Not specified	Not specified	Cross‐sectional	675	Gastric: 28.3% Colorectal: 50.5% Esophagus: 3.7% Small intestinal: 1.5% Hepatobiliary: 8.4% Pancreatic: 7.6%	Median: 60 (31–86)	Female: 35.1% Male: 64.9%	Not reported	Yes: 18.8% No: 81.2%	No
Lee 2016 [108]	Singapore	Not specified	Not specified	Qualitative	16	Breast	Majority 51–69	Female: 100%	Chinese: 75% Malay: 25%	Yes: 12.5% No: 87.5%	No
Leger 2025 [109]	United States	Not specified	Not specified	Qualitative	14	Prostate	Median: 65 (41–70)	Not reported	African American/Black: 100%	Yes: 14.0% No: 86.0%	No
Li 2010 [110]	China	Not specified	Not specified	Cross‐sectional	1156	Do not know: 4.2% Liver: 5.2% Lung: 42.0% Esophageal: 10.4% Stomach: 4.8% Colorectal: 7.3% Breast: 4.0% Pharyngeal: 6.1% Lymphoma: 7.4% Other: 8.7%	Patient: 46 Family: 53	Patient: Female: 37.9% Male: 62.1% Family: Female: 62.3% Male: 37.7%	Not reported	Yes: 4.8% No: 87.5%	Yes
Lu 2025 [111]	China	Not specified	Not specified	Qualitative	19	Liver	56.47 (SD: 10.20)	Female: 15.8% Male: 84.2%	Not reported	Yes: 47.4% No: 52.6%	No
Madsen 2002 [112]	Denmark	Not specified	Not specified	Cross‐sectional	88	Not reported	Not reported	Not reported	Not reported	Yes: 46.5% No: 53.5%	No
Madsen 2007 [113]	Denmark	Not specified	Chemotherapy	Qualitative	29	Breast: 82.8% Ovarian: 17.2%	53 (38–62)	Female: 100%	Not reported	Yes: 48.2% Declined: 51.8%	No
Madsen 2007 [114]	Denmark	Not specified	Chemotherapy	Qualitative	29	Breast: 82.8% Ovarian: 17.2%	53 (38–62)	Female: 100%	Not reported	Yes: 48.2% Declined: 51.8%	No
Maeng 2025 [115]	South Korea	Not specified	Not specified	Mixed methods	200	Not reported	Majority 50–59	Female: 50% Male: 50%	Not reported	Yes: 23.3% No: 76.7%	No
Mandane 2023 [116]	UK	Not specified	Not specified	Cross‐sectional	21	Endometrial	Not reported	Female: 100%	White: 71.4% Asian: 28.6%	Yes: 33.3% No: 66.7%	No
Manne 2015 [117]	United States	Not specified	Not specified	Cross‐sectional	1256	Not reported	58 (SD: 11.8)	Female: 100%	Caucasian: 88.3% Black: 9.2% Asian: 1.4% Native Hawaiian/Pacific Islander: 0.1% American Indian: 0.1% Mixed race: 0.6% Other: 1.0%	Not reported	No
Markan 2024 [118]	United States	Not specified	Not specified	Mixed methods	Survey: 194 Focus group: 28	Breast	Black: 55 (35–78) Non‐Black: 63 (27–88)	Female: 100%	Black: 25.8% Non‐Black: 74.2%	Yes: 10.8% No: 89.2%	No
McGrath‐Lone 2015 [119]	UK	Not specified	Not specified	Mixed methods	Survey: 66953 Interview: 25	Breast	Median: 58.7 (38–79)	Female: 100%	White: 76%	Yes: 40.0% No: 60.0%	No
McKinney 2021 [120]	United States	Not specified	Not specified	Cross‐sectional	21	Breast: 19.0% Colorectal: 19.0% Gastroesophageal: 14.3% Non‐small cell lung: 14.3% Pancreatic: 14.3% Ovarian: 9.5% Prostate: 4.8% Uterine: 4.8%	Not reported	Female: 66.6% Male: 33.4%	White: 80.9% African American: 9.5% American Indian or Alaska Native: 4.7% Other: 4.7%	No: 100%	No
Meropol 2007 [121]	United States	Not specified	Not specified	Cross‐sectional	170	Breast: 22% Lung: 16% Leukemia: 12% Colorectal: 10% Prostate: 8% Lymphoma: 7% Other: 26%	Median: 56 (22–85)	Female: 53% Male: 47%	White: 85% African American: 13% Black (not African descent): 0.5% Native American/Pacific Islander: 0.5% Haitian‐West Indian: 0.5% Missing: 0.5%	Not reported	No
Mobley 2023 [122]	United States	Not specified	Not specified	Qualitative	9	Acute lymphoblastic leukemia: 66.7% Germ cell tumor (testicular): 22.2% Acute promyelocytic leukemia: 11.1%	Majority 18–20	Female: 22.2% Male: 66.7% Transgender female: 11.1%	White: 55.6% Hispanic: 44.4%	Not reported	No
Mokhnatkin 2023 [123]	United States	Not specified	Not specified	Qualitative	32	Breast	Patients: Majority 70–79 Caregivers: Majority 50–59	Patient: Female: 100% Caregiver: Female: 62.5% Male: 37.5%	Patients: Race: White: 69% Black: 19% Asian: 6% Other: 6% Ethnicity: Hispanic or Latino: 19% Caregivers: Race: White: 56% Black: 19% Asian: 6% Others: 19% Ethnicity: Hispanic or Latino: 19%	Yes: 88.0% No: 12.0% Declined: 12.0%	Yes
Mooney‐Doyle 2025 [124]	United States	Not specified	Not specified	Mixed methods	21	I/GU: 14.3% Hematologic: 33.3% Lung: 33.3% Breast/GYN: 19.0%	58.52 (34–82)	Female: 57.1% Male: 42.9%	White: 90.5% Non‐white: 9.5%	Yes: 15.0% No: 85.0%	No
Moorcraft 2016 [125]	UK	Not specified	Various	Cross‐sectional	276	Colorectal: 42% esophagogastric: 36% Pancreatic: 7% Hepatobiliary: 4% Carcinoma of unknown primary/other GI: 1% Hodgkin's lymphoma: 4% Non‐Hodgkin's lymphoma: 6%	Median: 64 (19–85)	Female: 32% Male: 68%	White: 86% Asian: 8% Black: 3% Other: 3%	Yes: 88.0% Declined: 8.4%	No
Moreland 2024 [126]	United States	Not specified	Not specified	Cross‐sectional	300	Lung: 15% Breast: 10% Head and neck: 10% Colorectal: 8% Pancreatic: 8% Ovarian: 6% Prostate: 6% Endometrial: 5%	Median: 64 (18–93)	Women: 56% Men: 44%	White: 77% Non‐white: 23%	Yes: 27.0% No: 5.0% Declined: 73.0%	No
Moynihan, 2012 [127]	UK	Phase III	Not specified	Qualitative	24	Bladder	Accepters: 65 Decliners: 71 Accepters then decliners: 74	Female: 20.8% Male: 79.2%	Not reported	Yes: 50.0% Declined: 50.0%	No
Murrell 2026 [128]	United States	Not specified	Not specified	Mixed methods	98	Not reported	54.65 (SD: 11.37)	Female: 56.12% Male: 42.86% Intersex: 1.02%	White or Caucasian: 18.37% African American/Black: 69.39% Other: 7.14% Prefer not to say: 5.10%	Not reported	No
Murthy 2012 [129]	India	Phase III	Not specified	Randomized controlled trial	1355	Mouth	47.4	Female: 20.5% Male: 79.5%	Not reported	Yes: 37.0% Declined: 63.0%	No
Nguyen 2005 [130]	United States	Not specified	Not specified	Mixed methods	19	Not reported	(33–71)	Female: 100%	Asian American: 100%	Yes: 10.5% No: 89.5%	No
Paidipati 2023 [131]	United States	Multiple phases	Not specified	Qualitative	20	Pancreatic cancer: 20% Breast cancer: 20% Lung cancer or mesothelioma: 10% Bladder cancer: 5% Multiple myeloma: 15% Leukemia (Chronic Lymphocytic Leukemia, Acute Myeloid Leukemia, Acute Lymphoblastic Leukemia): 20% Lymphoma: 10%	Not reported	Female: 55% Male: 45%	Not reported	Not reported	Yes
Parikh 2022 [132]	United States	Not specified	Not specified	Qualitative	25	Breast	56.79 (31–71)	Female: 100%	Non‐Hispanic white: 64% Black: 12% Hispanic: 8% Asian: 12% American Indian: 4%	No: 100%	No
Park 2020 [133]	South Korea	Not specified	Not specified	Cross‐sectional	333	Colorectal: 35.4% Gastric:15.6% Lung: 10.5% Breast: 0.6% Pancreatobiliary: 5.7% Genitourinary:5.1% Sarcoma: 11.7% Neuroendocrine tumor: 5.7% Other rare tumors:9.6%	Median: 59 (29–70)	Female: 36.9% Male: 63.1%	Not reported	Yes: 19.0% No: 81.0%	No
Paskett 1996 [134]	United States	Not specified	Not specified	Qualitative	82	Breast	Majority < 65	Female: 100%	White: 58% African American: 42%	Yes: 17.0% No: 82.3%	No
Pearce 2018 [135]	UK	Not specified	Multimodal	Qualitative	21	Bone	Median: 20 (15–25)	Women: 33.3% Men: 66.7%	Not reported	Yes: 90.5% Declined: 28.6%	No
Penberthy 2012 [136]	United States	Not specified	Not specified	Cross‐sectional	1955	Majority 49–57 Solid tumor:81.7% Hematological: 15.3% Missing: 3.0%	56	Female: 100%	White: 64% African American: 36%	No: 43.4% Declined: 46.6%	No
Petitte 2019 [137]	United States	Not specified	Not specified	Qualitative	9	Breast: 100% Colon: 11.1%	(40–70)	Not reported	Not reported	Yes: 100%	No
Quinn 2007 [138]	United States	Not specified	Not specified	Qualitative	43	Lung	(42–74)	Female: 24.2% Male: 75.85	White: 86.0% African American: 9.3% Asian: 2.3% Hispanic: 2.3%	Yes: 41.9% No: 58.1%	No
Quinn 2012 [139]	United States	Not specified	Not specified	Qualitative	48	Lung cancer: 45.8% Breast cancer: 18.8% Hematological cancer: 12.5% Genitourinary cancer: 10.4% Head and neck cancer: 8.3% Did not disclose the cancer type: 12.5%	Mean: 66 (32–75)	Female: 37.5% Male: 62.5%	Not reported	Yes: 52.1% No: 27.1% Declined: 20.8%	No
Quinn 2013 [140]	US & Puerto Rico	Not specified	Not specified	Qualitative	36	Pelvic/Stomach: 11.1% Breast: 27.8% Prostate: 8.3% Colorectal: 8.2% Lymphatic: 5.6% Pulmonary: 5.6% Pancreas: 5.6% Ovarian: 5.6% Multiple Myeloma: 5.6% Brain: 5.6% Leukemia: 2.8% Bones and stomach: 2.8%	Majority 50+	Female: 75% Male: 25%	Hispanic: 100%	Not reported	Yes
Radecki Breitkopf 2016 [141]	United States	Not specified	Immunotherapy	Qualitative	72	Ovary	Patient: 59 (36–76) Family member: 56 (25–81)	Patient: Female: 100% Family member: Female: 59%	Patient: White: 94% Other (Mexican): 3% Other (Puerto Rican): 3% Family member: White: 95% Missing: 5%	Yes: 30.3%	Yes
Ramers‐Verhoeven 2014 [142]	US, UK, Germany, France, Italy, and Japan	Not specified	Not specified	Qualitative	120	Not reported	Not reported	Not reported	Not reported	Yes: 14.6% No: 66.7% Declined: 18.8%	Yes
Read 2009 [143]	Canada and US	Not specified	Not specified	Cross‐sectional	495	Not reported	Adolescent and young adult: Median: 18 (12–22) Parents Median: 40 (15–74)	Not reported	Not reported	Yes: 25.0%	Yes
Rezash 2020 [144]	United States	Early phase (I/I‐II)	Not specified	Mixed methods	38	Solid tumor	Patient: 57.58 (39–70) Caregiver: 55.63 (24–90)	Patient: Female: 47.4% Male: 52.6% Caregiver: Female: 63.2% Male: 36.8%	Not reported	Yes: 100%	Yes
Ridgeway 2017 [145]	United States	Not specified	Not specified	Qualitative	72	Ovary	Patient: 59.2 (36–76) Family member: 56.0 (25–81)	Female: 100%	Patient: White, non‐Hispanic: 93.9% Family member: White, non‐Hispanic: 100%	Not reported	No
Riggan 2023 [146]	United States	Not specified	Not specified	Qualitative	61	Breast and ovary	Patient: 59.5 (31–86) Family member: 46.3 (29–66)	Female: 100%	African American/Black: 100%	Yes: 24.5%	No
Rivera‐Díaz 2020 [147]	Puerto Rico	Not specified	Not specified	Qualitative	34	Breast	Not reported	Female: 100%	Not reported	Yes: 32.4% No: 67.6%	No
Sabesan 2011 [148]	Australia	Not specified	Not specified	Cross‐sectional	178	Not reported	56 (SD: 11.9)	Women: 59.8% Men: 40.2%	Not reported	No: 100%	No
Salem 2019 [149]	Lebanon	Multiple phases	Not specified	Qualitative	200	Breast: 35% Central nervous system: 1% Gastrointestinal: 19% Genitourinary: 10% Head and neck: 2% Hematologic malignancies: 28% Thoracic: 5%	Median: 51 (19–86)	Female: 61.5% Male: 38.5%	Lebanese: 90.5% Arab/non‐Lebanese: 9.5%	Yes: 3.5%	No
Sawyer 2021 [150]	UK	Early phase (I/I‐II)	Not specified	Qualitative	Focus group: 12 Interview: 22	Interview: Breast: 31.82% Colorectal: 13.64% Hematological: 9.09% Lung: 22.72% Lymphoma: 18.18% Penile: 4.55% Focus group: Colorectal:16.67% Head and neck: 8.33% Lung: 16.67% Leukemia: 8.33% Lymphoma: 41.67% Renal: 8.33%	Interview: Median: 65.5 (42–83) Focus group: Median: 68.5 (52–79)	Interview: Female: 59% Male: 41% Focus group: Female: 8% Male: 92%	Interview: White British: 95% Chinese: 5% Focus group: White British: 100%	Not reported	No
Shah 2012 [151]	United States	Not specified	Radiotherapy	Qualitative	46	Prostate	Median: 65	Male: 100%	White: 72% Black: 28%	Not reported	No
Shnorhavorian 2018 [152]	United States	Not specified	Not specified	Cross‐sectional	515	Acute lymphocytic leukemia: 4.1% Germ cell tumor: 39.0% Hodgkin lymphoma: 27.4% Non‐Hodgkin lymphoma: 24.9% Sarcoma: 4.7%	Majority 25–29	Female: 36.5% Male: 63.5%	White, Hispanic: 21% White, non‐Hispanic: 59% Black: 8.5% Asian/Pacific Islander: 9.7% Other: 1.7%	Yes: 6.0%	No
Slevin 1995 [153]	UK	Not specified	Not specified	Cross‐sectional	75	Gastrointestinal tract: 20% Lymphomas:20% Breast: 20% Ovary: 11% Seminomas: 11% Lung: 6% Myeloma: 3% Various other sites: 9%	50.1 (17–83)	Female: 52% Male: 48%	Not reported	Not reported	No
Smith 2024 [154]	United States	Not specified	Not specified	Cross‐sectional	385	Breast	Median: 52 (25–85)	Cisgender female: 72.2% Cisgender male: 0.3% Chose not to disclose or missing: 27.5%	Race: White/Caucasian: 72.5% Black/African American: 2.6% Other race/more than one race: 4.7% Missing: 20.3% Ethnicity Hispanic: 3.6% Non‐Hispanic: 78.4% Missing: 17.9%	Yes: 18.4% No: 73.8%	No
Staniszewska 2018 [155]	Poland	Not specified	Not specified	Cross‐sectional	256	Not reported	46 (21–77)	Women: 54% Men: 46%	Not reported	No: 100%	No
Stephen 2026 [156]	UK	Not specified	Not specified	Mixed methods	768	Breast	Majority 51–60	Female: 99.6% Male: 0.3% Not answered: 0.1%	White UK: 92.2% Irish: 1.2% Gypsy or Irish Traveler: 0.1% White and Black Caribbean: 0.7% White and Black African: 0.1% White and Asian: 0.4% Indian: 0.8% Chinese: 0.1% African: 0.4% Any other Asian background: 0.1% Any other mixed background: 0.4% Any other white background: 3.3% Any other ethnic group: 0.1% Not answered: 0.1%	Yes: 13.9% No: 83.5%	No
Stevens 2004 [157]	UK	Not specified	Not specified	Qualitative	22	Breast	Median: 58 (30–78)	Female: 100%	White: 100%	Yes: 14.7% No: 85.3% Declined: 16.2%	No
Sun 2023 [158]	China	Not specified	Not specified	Cross‐sectional	211	Not reported	58.60 (SD: 12.95)	Female: 48.3% Male: 51.7%	Not reported	Not reported	No
Sutherland 1998 [159]	Canada	Not specified	Not specified	Cross‐sectional	150	Head and neck 42% Breast:14% Other: 44%	55.9 (19–82)	Female: 47% Male: 53%	Not reported	No: 100%	No
Thorne 2013 [160]	Canada	Not specified	Not specified	Qualitative	22	Breast: 50.0% Prostate: 9.1% Lung: 9.1% Melanoma: 9.1% Gynecological: 4.5% Gastrointestinal: 4.5% Abdominal: 4.5% Pancreatic: 4.5% Hematologic: 4.5%	(32–75)	Women: 72.7% Men: 27.3%	Caucasian: 86% Asian/South Asian: 14%	Not reported	No
Townsley 2006 [161]	Canada	Not specified	Not specified	Mixed methods	Survey: 94 Interview: 17	Skin (not melanoma): 14% Melanoma: 4% Lung: 5% Breast: 18% Prostate: 20% Colon/rectum: 29% Lymphoma: 9% Other: 31%	Majority 70–74	Female: 45% Male: 54% NA: 1%	Not reported	Yes: 23.0% No: 74.0%	No
Tu 2005 [162]	United States	Not specified	Not specified	Qualitative	34	Not reported	53.3 (36–80)	Female: 94.1% Male: 13.5%	Chinese: 100%	Yes: 2.9% No: 97.1% Declined: 11.8%	Yes
Tyne 2025 [163]	Australia	Early phase (I/I‐II)	Not specified	Qualitative	3	Leiomyosarcoma: 33.3% Intrahepatic cholangiocarcinoma: 33.3% Advanced pancreatic cancer: 33.3%	Not reported	Not reported	Not reported	Yes: 100%	No
Ulrich 2022 [164]	United States	Multiple phases	Not specified	Cross‐sectional	334	GI or GU: 35.0% Hematologic‐lymphatic: 31.4% Lung: 15.3% Breast or gynecological: 18.3%	61.9 (SD: 11.5)	Female: 52.1% Male: 47.9%	White: 86.2% Other: 13.8%	Yes: 100%	No
van Gurp 2022 [165]	the Netherlands	Early phase (I/I‐II)	Not specified	Qualitative	13	Gastro‐intestinal: 38.5% Hepatobiliary/pancreatic: 23.1% Gynecological: 15.4% Lung: 15.4%	60.5 (32–69)	Female: 46.2% Male: 53.8%	Not reported	Yes: 15.4% Declined: 23.1%	No
Virani 2011 [166]	United States	Not specified	Not specified	Cross‐sectional	230	Skin: 27.3% Breast: 23.5% Prostate/bladder: 9.5% Lymphoma: 7.3% Leukemia: 5.2% Ovarian: 5.2% Lung: 4.7% Colorectal: 3.5% Head and neck: 3.5% Endometrial: 2.6% Testicular: 1.3% Other: 6.1%	Majority 55–64	Female: 62.2% Male: 37.8%	White: 79.1% Native American: 13.9% Other/Unknown: 5.6% African American: 0.8% Hispanic/Latino: 0.4%	Yes: 9.1% No: 90.9%	No
Weckstein 2011 [167]	United States	Not specified	Not specified	Cross‐sectional	287	Breast: 31.4% Lung: 12.8% Colorectal: 10.9% Hematologic: 9.6% Prostate: 8.1% Genitourinary, non‐prostate: 5.4% Head and neck: 3.8% Gynecologic: 3.7% Gastric/esophageal: 2.4% CNS: 2.4% Pancreas: 2.0% Other: 7.5%	Majority 56–65	Not reported	Not reported	Yes: 25.8% No: 24.0% Declined: 41.8%	No
Wenzel 2015 [168]	United States	Not specified	Not specified	Qualitative	32	Breast: 62.5% Prostate: 28.1% Colorectal: 6.3% Lung: 3.1%	Acceptors: Median: 58 (32–77) Decliners: Median: 55 (41–73)	Female: 62.5% Male: 37.5%	African American/Black: 100%	Declined: 56.3%	No
Wilhøft Kristensen 2024 [169]	Denmark	Not specified	Radiotherapy	Qualitative	14	Laryngeal and pharyngeal	(53–75)	Female: 42.9% Male: 57.1%	Not reported	Not reported	No
Williams 2024 [170]	United States	Not specified	Not specified	Cross‐sectional	650	Breast: 59% Hematologic: 31% Other: 9%	Median: 57	Female: 84% Male: 16%	Non‐Hispanic White: 52% Black/Hispanic/Other: 48%	Yes: 18.0%	No
Williams 2025 [171]	Australia	Not specified	Not specified	Qualitative	16	Ovary	57 (44–74)	Female: 100%	Not reported	Not reported	No
Williams 2026 [172]	Australia	Not specified	Not specified	Mixed methods	272	Ovary	61.5 (18–89)	Female: 100%	Australian: 90.5% Other: 7.8% Prefer not to say: 1.7%	Yes: 26.1% No: 66.5%	No
Wong 2016 [173]	United States	Not specified	Not specified	Cross‐sectional	1233	Breast: 25.7% Colorectal: 10.2% Lung: 14.3% Pancreatic: 5.0% Prostate: 8.4% Other: 36.0%	Majority < 65	Female: 58.5% Male: 41.5%	White: 88.24% Non‐white: 11.52% Missing: 0.24%	Not reported	No
Woodgate 2010 [174]	Canada	Not specified	Not specified	Qualitative	31	Leukemia or lymphoma: 73% Solid tumor: 27%	(3–17)	Female: 64.5% Male: 35.5%	White: 87.1% Aboriginal: 12.9%	Not reported	Yes
Yu 2026 [175]	China	Not specified	Not specified	Mixed methods	11	Pancreatic: 54.5% Gastric: 18.2% Rectal: 9.1% Colon: 9.1% Cervical: 9.1%	57.73 (28–77)	Female: 36.4% Male: 63.6%	Not reported	Not reported	No
Zdenkowski 2019 [176]	Australia	Not specified	Not specified	Cross‐sectional	188	Breast: 44.7% Lung: 5.3% Prostate: 8.0% Colorectal: 16.5% Other: 25.5%	59.8 (SD: 13.3%)	Female: 67.0% Male: 32.4% Missing: 0.5%	Not reported	Yes: 19.7% No: 79.8%	No
Zyumbileva 2026 [177]	France, Greece, Netherlands, Poland, Slovakia, Slovenia, Spain, Sweden, UK, Brazil, Bulgaria, Italy, Mexico, and Portugal	Not specified	Not specified	Qualitative	11	Breast	49 (SD: 12.2)	Female: 100%	Not reported	Yes: 18.0% No: 73.0%	No

^a^ For age, the mean (range) is reported unless otherwise specified; SD: standard deviation.

^b^ Sex/gender is reported as female/male, as several studies did not distinguish between sex and gender and/or used the terms interchangeably.

^c^ Trial experience is reported as “yes” if the participant had taken part or agreed to take part in a clinical trial.

### 3.1 Psychosocial Barriers

All but five studies reported psychosocial barriers related to personal beliefs, preferences, and emotional needs (*n* = 126; 96.1%) [72, 145, 163, 170, 171]. This included fear, distrust, a lack of family or physician support, and emotional distress. Two studies did not further specify the type of psychosocial concerns, and instead described them as general bad feelings, doubt, or fear of participating [81, 122].

#### 3.1.1 Fear

##### 3.1.1.1 Fear of Adverse Events

Fear of side effects or adverse reactions to experimental treatments was reported in 61.8% of studies (*n* = 81) [25, 26, 27, 40, 52, 53, 56, 57, 58, 61, 62, 64, 65, 66, 67, 68, 69, 70, 71, 73, 74, 75, 78, 79, 82, 86, 89, 91, 92, 93, 95, 96, 97, 99, 100, 101, 102, 103, 105, 110, 111, 113, 114, 115, 117, 118, 121, 123, 124, 127, 128, 129, 130, 131, 132, 133, 135, 136, 137, 140, 141, 147, 150, 151, 152, 154, 155, 156, 158, 159, 161, 164, 165, 166, 167, 168, 169, 172, 174, 175, 177]. Prevalence of such fear across studies varied widely, from 8.7% [136] to 94% [103], reflecting differences in whether side effects were considered a primary or hypothetical reason for refusal. Two studies found that among trial participants, 41%–48% reported experiencing or having concern with bothersome side effects [95, 164]. Concerns about adverse events were higher among trial decliners and non‐enrolled patients, older adults, and certain ethnic groups including Hispanic patients compared to White patients and non‐Black women compared to Black women [25, 26, 61, 70, 78, 93, 118].

##### 3.1.1.2 Fear of Being Experimented on

This theme captured fear of being experimented on or being used as a “guinea pig” or “lab rat”. These concerns were reported in 51.9% of studies (*n* = 68) [25, 26, 40, 52, 53, 55, 58, 59, 60, 61, 62, 64, 65, 66, 68, 69, 70, 75, 76, 78, 79, 82, 83, 85, 87, 89, 90, 91, 93, 95, 96, 97, 99, 100, 103, 104, 105, 109, 110, 111, 112, 117, 120, 121, 127, 128, 130, 134, 135, 136, 138, 139, 140, 142, 143, 146, 147, 148, 150, 151, 152, 155, 157, 158, 162, 164, 166, 177], though prevalence varied widely across quantitative studies. Tu et al. reported that among female Chinese American cancer patients, 71% held negative perceptions about trials including fear of being “treated like a guinea pig” [162]. Conversely, among individuals with trial experience in Jiang et al. and Ulrich et al., only 3.5%–5.6% reported feeling like “guinea pigs”, or that others perceived them as guinea pigs [95, 164]. Findings were inconsistent in terms of whether participant sub‐groups reported experimentation as a major concern. For example, while two studies reported higher concern among Black patients compared to White or Hispanic patients [26, 91], Penberthy et al. reported that more White participants listed fear of experimentation as a refusal reason compared to African American participants [136].

##### 3.1.1.3 Fear of Randomization

Fears related to the randomization process were presented in 54 studies (41.2%) [53, 54, 55, 57, 58, 60, 61, 64, 65, 68, 71, 76, 77, 79, 83, 84, 86, 87, 88, 89, 90, 92, 93, 94, 100, 105, 107, 108, 109, 112, 113, 114, 117, 120, 121, 123, 127, 128, 130, 134, 135, 136, 137, 138, 149, 151, 152, 153, 160, 164, 167, 169, 172, 175]. Participants in 22 studies were specifically fearful of receiving a placebo or “sugar pill” [53, 54, 55, 57, 58, 60, 62, 71, 83, 85, 88, 89, 91, 105, 109, 120, 123, 130, 138, 149, 164, 172]. Avis et al. reported 44.7% of trial decliners agreed that random assignment would discourage participation, compared to 12.4% of acceptors [61]. Conversely, Alhajji et al. found no significant difference between participants and non‐participants concerning fear of randomization or receipt of a sugar pill [55]. In a qualitative study with adolescent and young adult (AYA) patients and parents, participants expressed fear of decisional regret if the patient was randomized to an inferior treatment arm, and parents felt the blame would be on them [92]. Discomfort with randomization was common among older adults [65], rural versus inner city participants [83], and among White compared with African American participants [136].

##### 3.1.1.4 Fears Related to New or Experimental Treatments

Fears related to the interventional treatment's safety and efficacy compared to standard treatment were identified in 67 studies (57.9%) [40, 52, 56, 57, 58, 59, 60, 61, 62, 63, 64, 66, 68, 69, 70, 75, 77, 78, 79, 82, 84, 86, 87, 88, 89, 92, 93, 96, 97, 98, 100, 101, 103, 105, 107, 108, 110, 111, 113, 114, 116, 117, 119, 120, 126, 127, 128, 129, 134, 136, 139, 140, 141, 143, 144, 147, 148, 149, 151, 152, 155, 157, 161, 162, 167, 172, 175], with variation across sub‐groups. This type of fear was most prevalent among elderly participants in Townsley et al., where 90% of participants expressed concern of whether or not a new treatment works [161]. Differences were observed across sub‐groups, as three studies reported higher prevalence of fear of new treatments or preferences for standard care among racialized groups compared to White participants [70, 126, 136]. Additionally, Read et al. reported this concern was higher among parents of adolescent patients compared to the patients themselves [143].

##### 3.1.1.5 Fear of Lack of Personal Benefit

Beyond concerns about treatment safety, participants also reported fears related to lack of personal benefit in 37.4% of studies (*n* = 49) [40, 52, 55, 56, 58, 61, 67, 68, 70, 71, 74, 75, 78, 79, 86, 89, 90, 91, 92, 93, 98, 99, 102, 104, 105, 111, 115, 116, 117, 121, 123, 127, 128, 131, 132, 137, 138, 143, 144, 148, 152, 154, 156, 157, 158, 164, 166, 169, 175]. A total of 25 studies reported participants felt clinical trials would not guarantee the best health outcome or provide personal benefit, and might cause harm [40, 52, 55, 56, 58, 67, 70, 75, 79, 86, 89, 90, 92, 98, 104, 105, 116, 123, 128, 138, 144, 156, 164, 169, 175]. Some studies reported concern that trial participants would receive sub‐optimal care, though prevalence was low [55, 91, 93, 117, 128, 148, 154, 164]. Similarly, 13 studies reported concerns that clinical trial participation would lead to treatment delays [71, 79, 93, 111, 115, 116, 118, 123, 132, 137, 154, 158, 169]. As reported in a qualitative study among AYA's with trial experience, skepticism exist on whether trials are in the patient's best interest [52].

##### 3.1.1.6 Lack of Control

Fears owing to lack of control were reported in 48 studies (36.6%) [25, 27, 40, 51, 54, 64, 66, 67, 69, 72, 73, 74, 75, 76, 78, 79, 83, 86, 87, 93, 94, 96, 97, 99, 101, 102, 105, 108, 112, 113, 124, 125, 127, 131, 135, 138, 139, 141, 142, 148, 150, 151, 153, 154, 156, 157, 159, 164], often related to the inability to have their treatment chosen by themselves or their physician [55, 76, 79, 86, 87, 93, 94, 112, 113, 127, 147, 148, 151, 153, 154]. Though not necessarily a barrier to participation, 26 studies reported the burden of participants feeling they had no choice but to participate due to pressure from family, providers, or having exhausted standard treatment options [40, 51, 54, 69, 72, 73, 75, 78, 79, 83, 94, 97, 99, 101, 102, 105, 108, 124, 125, 127, 138, 141, 142, 156, 157, 159]. As such, some participants believed trials were only offered as a last resort. Others feared trial participation would cause loss of control of their personal lives and prevent them from engaging in their usual activities, which was important in their decision against participating [25, 27, 66, 67, 73, 96, 101, 135, 139, 150, 164].

#### 3.1.2 Distrust

##### 3.1.2.1 Trust in Research and Healthcare

Lack of trust as a barrier to trial participation was reported in 49 studies (37.4%) [40, 52, 53, 55, 57, 61, 67, 70, 77, 79, 85, 87, 88, 89, 90, 93, 94, 96, 99, 103, 104, 105, 106, 108, 111, 112, 113, 114, 117, 118, 119, 121, 122, 125, 126, 127, 130, 133, 138, 143, 146, 147, 148, 149, 154, 155, 160, 162, 164]. Most studies reported distrust in medicine and healthcare providers [52, 53, 57, 67, 70, 85, 89, 93, 94, 103, 113, 114, 117, 118, 121, 154, 155, 162], researchers and research institutes [52, 55, 61, 85, 89, 99, 103, 104, 111, 118, 126, 146], or pharmaceutical companies and trial funders [55, 67, 77, 113, 114, 117, 155]. A total of 22 studies specifically reported concerns that research is done for the benefit of researchers or physicians (i.e., financial or career benefit), which meant patient well‐being may be overlooked [40, 55, 61, 67, 70, 79, 87, 89, 90, 96, 103, 104, 105, 106, 112, 114, 117, 119, 127, 138, 148, 155, 157, 160, 164]. For example, 34% of trial participants and 23% of non‐participants in Madsen et al. agreed with the perception that medical research is primarily performed to promote doctor's careers [112]. Although prevalence was generally moderate to low across quantitative studies, 76.6% of refusers in Kong et al. believed regulations protecting research participant rights were inadequate [103]. Distrust was also observed when potential participants were approached by healthcare providers with whom they had no prior relationship [79, 157].

##### 3.1.2.2 Historical Abuse

Distrust resulting from historical abuse was described in 17 studies (13.0%), primarily among Black or Hispanic participants and older adults [53, 54, 55, 59, 70, 79, 85, 87, 88, 90, 109, 118, 128, 132, 146, 147, 168]. Participants cited examples such as the Tuskegee Syphilis study—where Black men enrolled in a prospective study were coerced into participation and left untreated after treatment (penicillin) was made available [178]—as well as mistrust due to personal and historical experiences of racism in medicine. Within qualitative or mixed methods studies, participants commonly discussed past mistreatment of Black individuals receiving medical care.

#### 3.1.3 Lack of Support and Discouragement

A total of 32 (24.4%) studies discussed discouragement or lack of familial or provider support as participation barriers [26, 52, 54, 57, 59, 62, 68, 70, 79, 86, 93, 98, 100, 102, 110, 117, 122, 129, 130, 131, 132, 134, 135, 136, 137, 139, 154, 161, 162, 166, 167, 168]. The highest prevalence was reported by Ayodele et al. and Townsley et al., where 94% of participants cited their doctor's recommendation against participation as a reason for refusal [62, 161]. Conversely, the prevalence of family discouragement as a participation barrier ranged from approximately 1%–44% in quantitative studies. In a qualitative study with AYA's, participants discussed providers going to parents first about trial decisions, and parents telling their children they could not participate as a barrier [122].

#### 3.1.4 Emotional Distress

A total of 41 (31.3%) studies described how one's emotional state impacts participation decisions [51, 55, 60, 63, 68, 70, 72, 74, 79, 85, 89, 91, 94, 96, 99, 108, 112, 113, 116, 117, 118, 119, 122, 125, 127, 131, 134, 135, 143, 145, 150, 154, 157, 160, 164, 168, 169, 171, 172, 174, 177], such as if discussions about trial availability were raised too soon after diagnosis or during a stressful period [60, 63, 68, 74, 89, 96, 113, 119, 122, 134, 135, 136, 143, 145, 154, 157, 169, 171, 174]. Participants also described feeling overburdened by the amount of information presented [63, 68, 72, 85, 94, 99, 112, 118, 125, 127, 160, 164, 168, 169], and distress caused by their cancer diagnosis limiting their willingness and ability to seek or process trial‐related information [55, 79, 91, 116, 117, 122, 157, 177].

### 3.2 Logistical Barriers

Logistical challenges were raised in most studies as travel, financial, time, employment, family, or continuity of care burdens (*n* = 96; 73.3%) [25, 26, 27, 40, 52, 54, 55, 56, 57, 58, 59, 60, 61, 62, 63, 64, 65, 66, 67, 69, 70, 71, 72, 73, 74, 75, 77, 78, 79, 80, 81, 82, 84, 85, 89, 91, 93, 95, 96, 97, 98, 99, 101, 103, 104, 107, 108, 109, 111, 112, 115, 116, 117, 118, 120, 121, 123, 124, 127, 128, 129, 130, 131, 132, 133, 134, 136, 137, 139, 140, 143, 144, 147, 148, 150, 151, 152, 154, 155, 156, 159, 162, 163, 164, 166, 167, 168, 169, 170, 171, 172, 173, 174, 175, 176, 177].

#### 3.2.1 Travel

Travel barriers to trial participation were reported in less than half of studies (*n* = 53; 40.6%) [25, 26, 27, 52, 54, 56, 58, 60, 61, 62, 64, 65, 70, 72, 73, 74, 78, 79, 80, 81, 84, 85, 89, 93, 97, 98, 104, 109, 115, 116, 117, 123, 124, 127, 130, 131, 143, 144, 148, 154, 155, 156, 159, 163, 164, 166, 167, 169, 170, 171, 172, 176, 179]. Avis et al. found that travel was a barrier reported by 58.7% of decliners compared to only 10.5% of accepters [61]. Examples of specific challenges included driving in poor weather among older adults and long or inconvenient travel among individuals living in rural or remote areas, who were more likely to view travel as an important factor in deciding to participate [25, 148]. In a qualitative study, caregivers described the logistical burden that was placed on them, including the demand of traveling to trial sites and potentially relocating to be closer to the cancer center [131].

#### 3.2.2 Out‐Of‐Pocket Costs

A similar number of studies reported concerns about out‐of‐pocket costs as a barrier to participation (*n* = 54; 41.2%) [25, 26, 27, 52, 54, 55, 56, 57, 58, 61, 66, 70, 75, 78, 79, 80, 81, 84, 85, 89, 91, 93, 95, 103, 104, 109, 111, 117, 120, 121, 124, 129, 132, 136, 140, 143, 144, 147, 148, 150, 152, 154, 156, 162, 163, 164, 166, 167, 168, 170, 172, 173, 176, 177]. Participants cited travel expenses and other participation‐related costs as barriers, with 54% of participants in Zdenkowski et al. reporting they would not join a trial if out‐of‐pocket costs were involved [176]. Concerns specifically about out‐of‐pocket treatment, testing, and procedure costs were reported in 27 studies, with variable prevalence [25, 26, 52, 55, 56, 58, 61, 70, 75, 78, 79, 81, 85, 89, 91, 93, 117, 124, 132, 140, 152, 154, 164, 167, 168, 170, 173]. For example, Byrne et al. reported that 85% of survey participants worried that insurance would not cover additional interventions [26], while 9% of survey participants in Dolinsky et al. expressed concern about insurance denial [78]. These concerns were generally higher among non‐participants compared to trial participants [55, 61]. However, among trial participants in Jiang et al., less than half (47.2%) reported they had received satisfactory compensation for their participation [95].

#### 3.2.3 Additional Visits, Tests, and Time Commitments

Participants described inconveniences related to the burden of frequent hospital study visits, tests, and appointments in 60 studies (45.8%) [27, 40, 52, 54, 55, 57, 59, 61, 63, 65, 66, 67, 69, 70, 71, 72, 73, 74, 75, 77, 79, 82, 84, 89, 93, 95, 96, 97, 98, 99, 107, 108, 109, 115, 116, 117, 118, 120, 123, 127, 128, 132, 133, 134, 139, 143, 144, 148, 150, 151, 154, 155, 156, 159, 163, 164, 167, 169, 174, 175]. This was broadly defined by 35 studies as burdensome time requirements, inconvenience, or disruptions to daily life [27, 40, 55, 57, 59, 61, 66, 67, 69, 71, 72, 74, 77, 79, 89, 95, 96, 97, 98, 109, 117, 118, 128, 132, 133, 139, 143, 148, 151, 154, 159, 163, 164, 167, 169]. Specifically, 40 studies reported extra procedures or tests [27, 52, 54, 61, 63, 65, 67, 70, 71, 73, 74, 77, 82, 84, 93, 108, 115, 117, 120, 123, 127, 132, 148, 163, 167, 174], additional hospital visits or appointments [54, 59, 71, 72, 73, 79, 97, 99, 107, 108, 115, 116, 120, 144, 150, 155, 156, 175], prolonged hospitalization [27, 52, 67, 74], or increased treatment length or trial duration [63, 70, 134, 151] as barriers to participation. Avis et al. noted how concerns about time requirements were more prevalent among trial decliners (58.7%) compared to accepters (10.5%) [61], and Read et al. reported higher prevalence among AYAs (45%) compared to parents and caregivers (13%) [143].

#### 3.2.4 Time Off Work

Concerns that trial participation would affect participants' ability to work were reported in 23 studies (17.6%) [25, 26, 27, 52, 54, 58, 71, 75, 79, 109, 116, 124, 130, 131, 147, 148, 154, 156, 163, 164, 168, 169, 177]. Loss of work hours as an important factor in participation decisions, particularly among racialized individuals [54, 116]. Some AYA's in Abrahão et al. reported having to quit their jobs to comply with treatment, which caused financial strain [52]. In contrast, this concern was less commonly reported among adults aged > 65 [25].

#### 3.2.5 Impact on Family

A total of 23 studies reported concerns about the impact of trial participation on family members (17.6%) [25, 26, 54, 58, 64, 67, 69, 75, 79, 85, 93, 101, 109, 118, 124, 130, 131, 132, 147, 148, 154, 164, 169, 177]. Participants expressed worries about relying on family or friends for care, arranging childcare or eldercare, and being unable to meet family responsibilities. Prevalence varied widely across populations, with Basche et al. reporting that 82.7% of older adults indicated requiring care from family or friends was a barrier [25], and Javid et al. reporting that only 13% of participants anticipated that participation would burden their family [93]. Caregivers with trial experience in Paidipati et al. described significant effort scheduling appointments and other care necessities, which was burdensome [131]. Some heterogeneity was seen across sub‐groups: Sabesan et al. reported that the need for family or friends to accompany participants to appointments was a greater concern for rural or remote living participants [148], and Markan et al. reported that 24% of Black women surveyed found it difficult to find time to participate and agreed that they could participate if they had help at home, compared to 18% of non‐Black women [118].

#### 3.2.6 Continuity of Care

Anticipated and experienced continuity of care interruptions were reported in 11 (8.4%) studies [25, 26, 58, 61, 64, 72, 73, 74, 112, 152, 176]. Participants were reluctant to change providers or receive care outside of their usual treatment setting. For example, Zdenkowski et al. reported that 57% of participants indicated that they would not join a trial if it required changing oncologists [176]. Similarly, some expressed fear of being abandoned by healthcare providers following trial completion [72, 73, 74].

### 3.3 Informational Barriers

Informational barriers were reported in 90 studies (68.7%) and captured challenges related to patients' understanding and access to clinical trial information [25, 26, 51, 52, 54, 55, 56, 57, 58, 59, 60, 61, 63, 65, 66, 67, 68, 69, 70, 71, 72, 73, 74, 78, 79, 81, 83, 85, 86, 87, 88, 89, 91, 93, 95, 97, 98, 99, 102, 103, 104, 106, 109, 112, 114, 115, 117, 118, 119, 121, 122, 123, 125, 127, 128, 129, 130, 133, 134, 135, 137, 138, 140, 141, 142, 143, 144, 145, 146, 147, 148, 149, 150, 152, 155, 156, 157, 158, 159, 160, 161, 162, 163, 166, 168, 169, 171, 172, 175, 177]. These barriers included limited awareness and information provision, misconceptions, difficulties with informed consent materials, and language barriers.

#### 3.3.1 Awareness of Available Trials

Barriers related to awareness of trials, defined as not knowing that relevant trials were available and how to identify or access participation opportunities, were reported in 27 studies (20.6%) [26, 55, 57, 58, 60, 66, 70, 78, 81, 85, 91, 104, 109, 115, 117, 121, 123, 133, 145, 146, 152, 156, 160, 161, 163, 171, 172]. Participants in 12 studies reported that their physicians did not discuss trials as an option with them [55, 58, 60, 70, 78, 79, 81, 91, 155, 160, 166, 171]. Those who sought trial information on their own found the search process confusing and overwhelming [109, 115, 145, 146, 156, 171]. Of the 25% of patients who had searched a trial registry in Stephen et al., 53% reported being unable to find the information they required [156]. Quantitative findings suggested that lack of awareness was common among specific populations, with one study reporting that 63% of AYA participants were unaware of available trials [152], and another indicating that Hispanic participants more frequently cited not knowing about trials as a barrier compared to White or Black participants [26]. In a qualitative study by Thorne et al., participants felt betrayed when they had not been offered a trial but later discovered that one may have been available [160].

#### 3.3.2 Limited Knowledge and Misconceptions About Clinical Trials

Limited knowledge or inaccurate beliefs about the purpose, procedures, design, or potential value of clinical trials were commonly reported (*n* = 57; 43.5%) [51, 52, 54, 56, 57, 59, 60, 61, 63, 66, 68, 70, 71, 74, 78, 79, 83, 85, 86, 87, 88, 89, 93, 95, 103, 106, 109, 112, 114, 117, 118, 121, 122, 123, 127, 130, 133, 134, 135, 137, 138, 140, 141, 142, 145, 147, 148, 149, 155, 156, 157, 158, 160, 161, 162, 175, 177]. Lack of understanding of trial objectives was particularly common, with one study reporting 94.2% of trial refusers lacked understanding of trial goal [103]. Misconceptions were also commonly described as confusion about the purpose of randomization, the concept of clinical equipoise, and doubts that participation would contribute to science or benefit others. The latter misconception varied between populations but was generally uncommon. For example, two studies reported that Black participants more often believed their communities had nothing to gain from research and disagreed that trials make a significant contribution to science [106, 118].

#### 3.3.3 Informed Consent and Study Materials

Barriers related to consent forms and trial documents were reported in 19 studies (14.5%), described as difficulty understanding study materials [51, 67, 85, 97, 99, 103, 109, 112, 115, 123, 125, 127, 140, 143, 150, 156, 157, 162, 171]. Across studies, participants described consent documents and trial information as lengthy, complex, or intimidating which caused challenges understanding trial procedures and, in some cases, feelings of being undermined during the consent process.

#### 3.3.4 Limited Information

A total of 47 studies (35.9%) described barriers related to inadequate trial information from healthcare providers and research team members [25, 51, 55, 56, 57, 59, 60, 63, 65, 69, 70, 72, 73, 74, 79, 81, 93, 97, 98, 99, 102, 106, 112, 115, 117, 118, 119, 122, 123, 125, 127, 128, 137, 142, 144, 145, 147, 155, 156, 157, 159, 161, 162, 166, 168, 171, 175]. Limited information referred to inadequate communication about trials in general and specific trials that they were approached about, which impacted informed decision making. Patients reported that clinical trials were not discussed or that information provided was unclear, insufficient, or not offered up front. In a cross‐sectional study, 77.5% of participants reported they received no information about trials, and 79.1% reported having no education about the benefits of trials [56]. As a result, some participants described needing to self‐advocate for information [171], however, participants in Chalela et al. and Kumar et al. felt too intimidated or did not feel free to ask questions [70, 106]. After trial participation, 64% of patients in Kearns et al. reported not receiving published results despite wanting them [97].

#### 3.3.5 Language

Language barriers were reported in 8 studies (6.1%), 5 of which were qualitative [70, 89, 122, 129, 130, 140, 162, 171]. An interview study of Asian American women reported that language was the second highest rated barrier, identified by over half of participants [130]. Most participants in this study had limited English proficiency, and few had oncologists who spoke their native language. Consistently, Quinn et al. noted a lack of trial information translated into Spanish, limiting accessibility for Hispanic participants [140].

### 3.4 Motivators, Facilitators and Recommendations

While the primary objective of this review was to identify barriers to cancer trial participation, we also report motivators and recommendations or facilitators for participation. Motivators for trial participation are reported in Figures 2, 3, 4. Recommendations or facilitators were discussed in 69 studies (52.7%) [26, 40, 52, 54, 56, 57, 59, 60, 61, 62, 63, 65, 66, 67, 68, 69, 70, 73, 74, 75, 85, 87, 88, 90, 92, 93, 95, 97, 103, 104, 105, 106, 108, 109, 115, 116, 118, 120, 121, 122, 126, 128, 130, 135, 136, 137, 138, 139, 141, 142, 144, 145, 146, 147, 150, 151, 154, 156, 157, 160, 161, 162, 163, 168, 171, 172, 176, 177].

**FIGURE 2:**
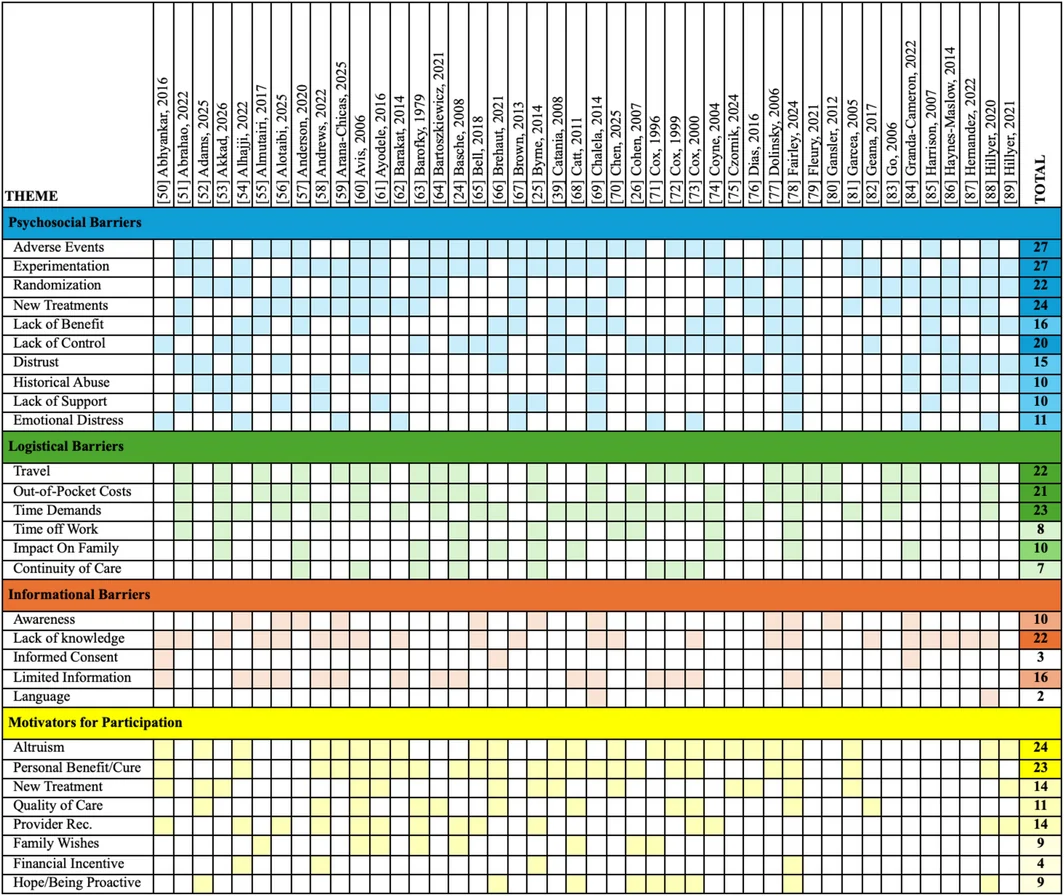
Heat map of patient and caregiver reported barriers and motivators.

**FIGURE 3:**
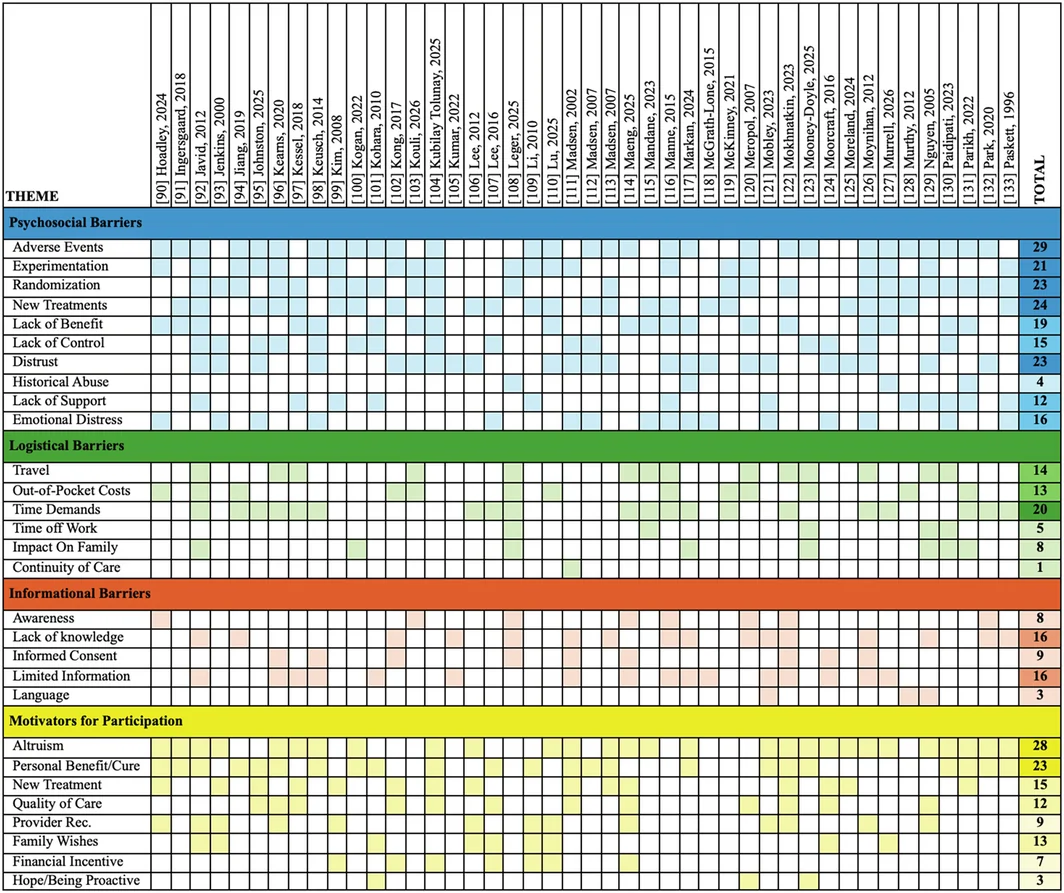
Heat map of patient and caregiver reported barriers and motivators (cont.).

**FIGURE 4:**
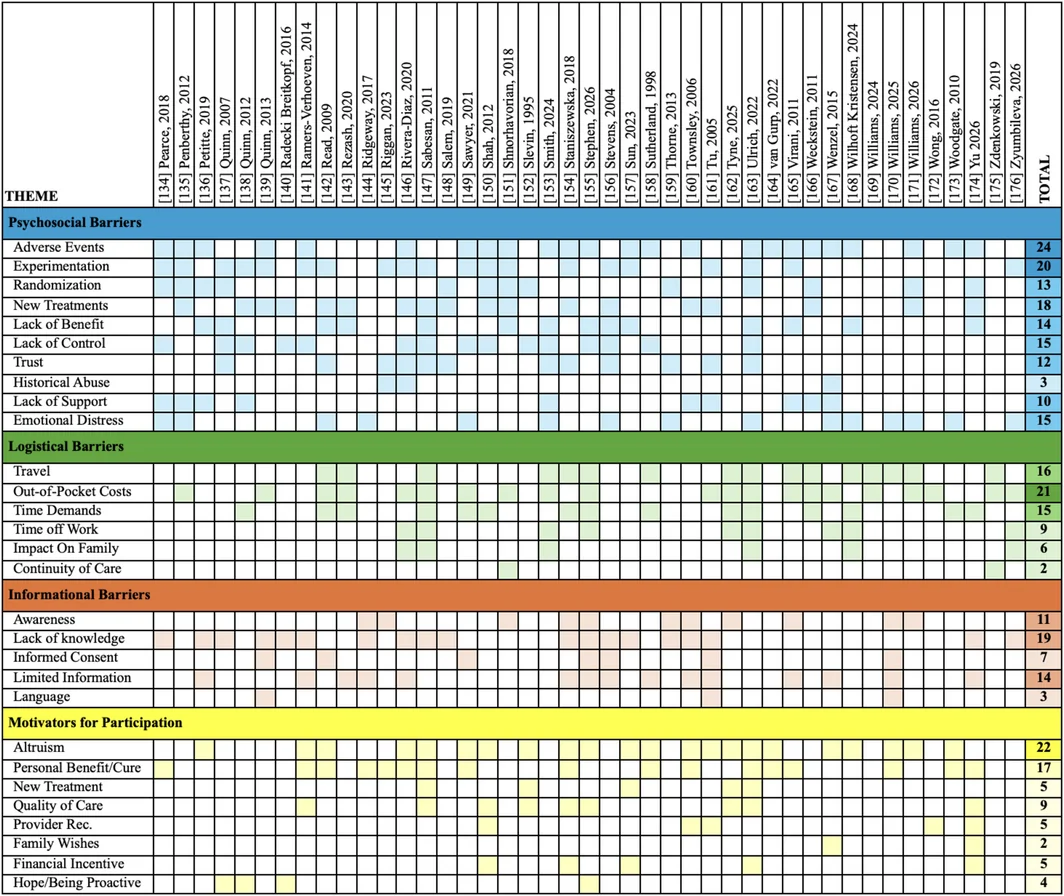
Heat map of patient and caregiver reported barriers and motivators (cont.).

The most commonly reported facilitators were those related to clear, accessible, and credible information, reported in 42.0% of studies (*n* = 55) [26, 40, 52, 54, 56, 57, 59, 60, 61, 63, 65, 66, 67, 69, 70, 73, 75, 79, 85, 87, 97, 99, 105, 106, 108, 109, 115, 116, 118, 120, 121, 122, 128, 135, 136, 137, 138, 139, 141, 142, 145, 146, 150, 151, 156, 157, 160, 161, 162, 163, 168, 171, 172, 177]. This included easily accessible information about available trials, as well as clear communication about the implications of participation. Participants recommended clearly written protocols and consent forms outlining rights, risks, requirements, and benefits in simplified language [52, 79, 93, 108, 109, 118, 150, 151, 171]. Several highlighted the importance of having their physician's input or advice [52, 67, 73, 74, 108, 109, 150, 151], being given sufficient time to ask questions and discuss trials with providers and research staff [56, 57, 60, 62, 63, 68, 87, 99, 105, 108, 116, 135, 139, 157, 160, 168], and opportunities to ask questions [68, 99, 157]. Information presented positively, concisely, and presented at appropriate times was similarly important [63, 65, 66, 85, 99, 115, 122, 157]. Some studies reported that access to trial data during and after participation could prevent misconceptions and help participants understand research impact [52, 67, 73, 79, 87, 136, 138, 171]. Others noted the importance of reducing language barriers [54, 162], and providing trial information in multiple formats (e.g., poster, emails, websites, videos) [52, 54, 57, 97, 106, 115, 145, 157, 168, 171, 172], and in multiple locations (e.g., hospitals, churches, schools) [52, 57, 79, 87, 146, 161]. Additionally, two studies suggested a centralized database of available trials [65, 156], and five recommended an advocate or navigator to help patients find available trials [88, 97, 115, 163, 171]. Finally, participants in two studies recommended opportunities for patients to involve their family members in decision‐making [66, 177].

The importance of building and maintaining trust was another facilitator for improved participation (22.9%; *n* = 30) [40, 52, 57, 59, 66, 69, 70, 79, 85, 92, 93, 95, 103, 105, 108, 115, 116, 118, 122, 130, 137, 144, 146, 147, 161, 162, 163, 171, 172, 177]. Participants agreed that having conversations about trials with a knowledgeable, respectful, and trusted healthcare provider would facilitate participation [52, 79, 118, 130, 137, 162, 171, 177], as well as having trust in medical staff, the healthcare system, and the research team [40, 57, 59, 69, 70, 85, 92, 93, 95, 103, 116, 118, 122, 137, 144, 147, 163, 172]. Participants in 8 studies felt that the treating physician should be the one to approach patients about trials, as they have already built a relationship with them [66, 92, 105, 108, 115, 137, 161, 162]. In the case of racialized groups, participants in five studies felt that sharing the same ethnicity as members of the research team was important [57, 85, 106, 109, 118].

Recommendations related to logistical support were also reported (*n* = 27; 20.6%) [26, 52, 54, 59, 65, 67, 75, 79, 90, 93, 95, 104, 106, 109, 115, 118, 126, 147, 150, 151, 154, 157, 162, 163, 172, 176, 177]. Reimbursement of expenses, compensation for participation, and clarity about potential costs were identified as a facilitator in 16 studies [26, 52, 54, 59, 65, 67, 79, 90, 95, 115, 118, 126, 137, 147, 151, 163], and participants in two studies indicated they would be more likely to join a trial if there were no out‐of‐pocket costs [162, 176]. Others reported that providing transportation and accommodations [26, 52, 54, 67, 79, 93, 104, 147], childcare, family support, or household assistance [26, 54, 79, 118, 150], opportunities for home, work, or telehealth visits [54, 79, 106, 154], and conducting tests, procedures, or treatments at a convenient location or at flexible times [52, 54, 57, 106, 109, 118, 154, 172, 176, 177] could ease participation burden. Finally, participants in two studies suggested using trial designs that give patients more autonomy [135, 157], and participants in 8 studies recommended providing mental health, spiritual, or peer supports during a trial [52, 57, 79, 115, 135, 137, 168, 177].

## 4 Discussion

We present a scoping review to synthesize patient and caregiver‐reported barriers and facilitators to participation in cancer clinical trials across diverse populations and study contexts. Notably, fear‐based (i.e., fear of adverse effects, experimentation, randomization) and informational barriers (i.e., limited knowledge about trials, limited information access) were commonly reported and often co‐occurred, highlighting the potential interplay between limited understanding and concerns about experimental treatments [180, 181, 182, 183]. This relationship may further be explained by health literacy, as trial awareness and participation are higher among more educated individuals [180, 184, 185]. Understanding whether fears stem from a lack of information is important, as these barriers may then be mitigated by providing clear, accessible information about trials. In addition, the overlap among informational, fear‐based, psychosocial, and structural barriers suggests these factors may be interconnected. These findings may provide a foundation for future research to develop and refine a conceptual framework describing how barriers interact to influence cancer clinical trial participation.

Though our review offers some insight into how barriers may differ between sociodemographic groups, such as distrust related to historic abuse, it highlights a lack of evidence on barriers specific to marginalized populations. While 11 studies included predominantly non‐White participants, few compared barriers across racial or ethnic groups, and their rationale behind these comparisons was missing. Previous literature has demonstrated lower participation among non‐White versus White patients [186, 187, 188]. Lower participation rates may reflect disparities in access to healthcare experienced by marginalized groups [189, 190, 191]. Evidence suggests that minority populations are less likely to be offered trials despite willingness to participate [192], as they are more often treated at under‐resourced hospitals and are more likely to be ineligible due to higher rates of comorbidities [7]. Additionally, registered trial protocols rarely mention translation services, which may limit access for non‐English speaking populations [193]. Included studies also lacked comparisons between patient‐reported barriers and other sociodemographic factors known to impact healthcare access, such as geographical location, income, and education [194, 195, 196, 197]. In addition, the majority of the identified studies were published in the United States, which may limit the transferability of findings to other settings. Future research is needed to understand how these barriers differ across geographic and socioeconomic groups to inform targeted recommendations that increase participation among marginalized populations [198].

The studies identified in our search were heterogeneous in terms of study design, with many lacking specificities regarding trial phase (83.2%) and intervention type (87.0%). Consequently, the findings provide a broad overview of patient and caregiver‐reported barriers to cancer clinical trial participation but cannot establish whether particular barriers are more prominent in certain trial phases, intervention types, or treatment settings. Further research is needed to elucidate recommendations tailored to specific trial phases and intervention types. In addition, future research would benefit from examining barriers unique to trial context, phases, and intervention types to support more targeted recruitment and retention strategies.

Finally, our findings reaffirm the paucity of evidence regarding caregiver‐reported barriers, with fewer than 10% of the included studies capturing their perspectives. This represents a significant gap, given the central role that caregivers play in patient care across all ages, particularly in pediatric settings where their involvement is integral to decision‐making and treatment support. Caregivers support clinical decision‐making and facilitate access to care, including transportation, medication adherence, and managing practical responsibilities [199, 200, 201]. Recommendations to improve clinical trial design must include the caregiver voice to capture the barriers they uniquely experience and recognize their critical role in facilitating patient participation.

### 4.1 Toward Improved Participation

Few studies offered concrete recommendations to mitigate participation barriers. In addition, recommendations were loosely defined and not specifically mapped to barriers, making it difficult to identify which challenges they would address. Critically, overlap between fear‐based and informational barriers suggests that addressing these concerns is essential to improving participation. Barriers related to trial randomization are especially challenging, as these elements are often fundamental to trial design. However, informational interventions, including web‐based education programs, decision aids, and tools designed to foster communication have demonstrated success in improving knowledge, attitudes, and informed decision making [202, 203, 204, 205, 206]. Such interventions have been shown to increase knowledge about trial purpose, ethics, and procedures, leading to more positive attitudes toward participation [43, 207, 208, 209]. In addition, providing simplified study materials (i.e., consent forms) with plain language and visual aids has yielded increased understanding of trial purposes and procedures across health literacy levels [210].

Consistent with our findings, recommendations have been made to tailor recruitment strategies to enhance participation of culturally and linguistically diverse (CALD) communities [211, 212]. A review and expert consultation in Australia identified three pillars to engage CALD populations: co‐designing trials with CALD community members, building trust by developing relationships with target communities, and investing sufficient time and resources to implement culturally responsive approaches such as staff training, translating study materials, providing in‐depth explanations of trials for patients, and fostering connections [213]. Where distrust drives hesitancy to participate in trials, researchers must prioritize transparency, respect, and relationship building. Strategies include engaging community members in trial design, having trusted providers conduct recruitment conversations, and providing transparent information on trial procedures, risks and benefits, data use, patient rights, and ethical commitments [87, 209, 214, 215, 216].

Recommendations have also been made to build solutions addressing logistical challenges directly into trial designs including transportation and lodging, reimbursing all travel‐related costs, and providing schedules well in advance [217, 218, 219]. Several initiatives have facilitated transportation, especially for socially vulnerable populations [220, 221, 222]. Researchers are also advised to provide transparent estimates about expected costs and timely reimbursement [217, 218, 219, 223]. Where reimbursement is not feasible, patients can be connected to organizations that provide financial support [224, 225, 226, 227, 228, 229]. In Canada, ULink and Hope Air assist patients with travel, lodging, and related expenses for clinical trials [228, 229]. In‐person visits can be minimized by conducting procedures (i.e., consent, check‐ins) virtually or at local community clinics [217, 219, 230, 231, 232, 233, 234, 235]. The Canadian Remote Access Framework for Clinical Trials (CRAFT) provides guidance for delivering trials to patients in remote and rural areas using satellite centers [236]. Recommendations include training for staff satellite centers, leveraging pre‐existing telemedicine infrastructure, and engaging rural clinicians and patients in clinical trial design. Initiatives that have begun successfully offering clinical trial procedures closer to home include the Pediatric Oncology Group of Ontario (POGO) Satellite Clinic Program and the Clinical Oncology Society of Australia (COSA) Australasian Tele‐Trial Model [237, 238]. Together, these approaches illustrate how flexible and patient‐centered trial delivery can mitigate many practical barriers identified in this review.

Despite ongoing efforts, clinical trials continue to fall short of meeting patient needs [24]. Future research must draw on patient and caregiver insights to guide the development of standardized, accessible, and patient‐centered designs. Integrating trials into the continuum of cancer care and clearly communicating them as a potential treatment option may improve access and uptake. Given the descriptive nature of this review, future research should examine the relative importance and causal influence of barriers, how they impact trial participation, which strategies are most effective in addressing them, and how these relationships vary across populations, trial types, and healthcare contexts.

### 4.2 Limitations

Our results should be interpreted in light of limitations. The review was limited to English‐language studies, which may have excluded relevant perspectives. Heterogeneity in study design, outcomes measurement, and reporting of the included studies contributed to the variability of findings. However, we present the results in the form of a heat map and prevalence estimates, providing a descriptive overview of how frequently each barrier and recommendation was identified across the included literature. This helps to illustrate the breadth and recurrence of the identified barriers and highlight areas that have received greater attention in the existing evidence base.

Several studies did not report key population characteristics, such as trial phase, race/ethnicity, and cancer type or stage, or stratify the reporting of the results by these factors, limiting the ability to draw conclusions on potential differences in barriers across groups. Although studies from all countries were included, differences in healthcare systems and their potential influence on barriers to clinical trial participation were not systematically examined.

Finally, the predominance of U.S.‐based studies may limit the transferability of findings to other regions. Included studies were not critically appraised; however, this review aimed to map the evidence rather than assess methodological quality consistent with JBI guidance [46].

## 5 Conclusion

Patient participation in cancer clinical trials is influenced by a range of psychosocial, informational, and logistical barriers that likely intersect with broader structural and social inequities. Our findings highlight the need for a better understanding of how experiences differ across sociodemographic groups and to uncover the mechanisms underlying these barriers. The findings of this scoping review will inform future research priorities and support the development of patient‐centered clinical trial frameworks to improve equitable access, accrual, and adherence to cancer clinical trials.

## Author Contributions

**Sarah Scruton:** conceptualization, investigation, writing – original draft, writing – review and editing, methodology, visualization, formal analysis, project administration, data curation. **Chisato Ito:** data curation, writing – review and editing, methodology, visualization, investigation. **Julia Bohnen:** data curation, writing – review and editing, investigation. **Robin Urquhart:** conceptualization, writing – review and editing. **Chiquita Hessels:** conceptualization, writing – review and editing. **Summer Konechny:** conceptualization, writing – review and editing. **Rosilene Kraft:** conceptualization, writing – review and editing. **Randy Thompson:** conceptualization, writing – review and editing. **Cheryl Ho:** conceptualization, writing – review and editing. **Deirdre Weymann:** conceptualization, writing – review and editing. **Yvonne Bombard:** conceptualization, writing – review and editing. **Emanuel Krebs:** writing – review and editing. **Avram Denburg:** conceptualization, writing – review and editing. **Timothy P. Hanna:** conceptualization, writing – review and editing. **Howard J. Lim:** conceptualization, writing – review and editing. **Saadia Shareen:** writing – review and editing, project administration, formal analysis, data curation. **Dean A. Regier:** conceptualization, writing – review and editing. **Samantha Pollard:** conceptualization, investigation, funding acquisition, writing – original draft, writing – review and editing, methodology, supervision.

## Funding

This work was supported by The Terry Fox Research Institute and Health Canada as a part of the Marathon of Hope Cancer Centres Network Patient Voices Initiative. The views expressed herein do not necessarily represent the views of Health Canada.

## Conflicts of Interest

Scruton, Ito, Bohnen, Urquhart, Hessels, Denburg, Thompson, Krebs, and Shareen have no conflicts of interest to declare. Hanna is the Ontario Health Cancer Care Ontario Radiation Oncology Clinical Quality Lead for the Radiation Treatment Program. Weymann and Pollard co‐direct IMPRINT Research Consulting and have consulted for AstraZeneca, Birota Economics Group, and LHS Labs. Regier reports that BC Cancer has received research funding for a project from Roche Canada.

## Supporting information


**Appendix 1:** Search strategies.
**Appendix 2:** Additional themes identified in the included studies.

## Data Availability

The data presented in this study are derived from the manuscripts cited in the reference list.
