# Supplementary material for: Patient and Caregiver Reported Barriers to Cancer Clinical Trial Participation: A Scoping Review
Source: Cancer Rep (Hoboken). 2026 Oct 13;9(10):e70700. doi: 10.1002/cnr2.70700 (PMC13662225; doi:10.1002/cnr2.70700)
Supplement: Supplementary file 1 [file CNR2-9-e70700-s001.docx]

**Appendices**

**Appendix 1. Search strategies**

**Appendix 2. Additional themes identified in the included studies**

**Appendix 1. Search strategies**

**PubMed**

("attitude to health"[MeSH Terms] OR "patient* attitude*"[Title/Abstract] OR "health knowledge, attitudes, practice"[MeSH Terms] OR "patient preference"[MeSH Terms] OR "patient* preference*"[Title/Abstract] OR "choice behavior"[MeSH Terms] OR "barrier*"[Title])

AND

("clinical trials as topic"[MeSH Terms] OR "clinical trial*"[Title/Abstract] OR "randomized controlled trial*"[Title/Abstract] OR "randomized controlled trials as topic"[MeSH Terms])

AND

("cancer"[Title/Abstract] OR "neoplasms"[MeSH Terms] OR "oncology"[Title/Abstract])

**Ovid MEDLINE**

| # | Query |
| --- | --- |
| 1 | Attitude to Health/ |
| 2 | patient* attitude*.ti,ab. |
| 3 | Health Knowledge, Attitudes, Practice/ |
| 4 | Patient Preference/ |
| 5 | patient* preference*.ti,ab. |
| 6 | Choice Behavior/ |
| 7 | barrier*.ti. |
| 8 | 1 or 2 or 3 or 4 or 5 or 6 or 7 |
| 9 | exp Clinical Trials as Topic/ |
| 10 | clinical trial*.ti,ab. |
| 11 | Randomized Controlled Trials as Topic/ |
| 12 | randomized controlled trial*.ti,ab. |
| 13 | 9 or 10 or 11 or 12 |
| 14 | Neoplasms/ |
| 15 | cancer.ti,ab. |
| 16 | oncology.ti,ab. |
| 17 | 14 or 15 or 16 |
| 18 | 8 and 13 and 17 |

**CINAHL Complete**

(MH "Attitude to Health" OR XB (patient* attitude*) OR MH "Attitude to Health" OR MH "Patient Preference" OR XB (patient* preference*) OR MH "Decision Making, Patient" OR TI (barrier*))

AND

(MH "Clinical Trials+" OR XB (clinical trial*) OR MH "Randomized Controlled Trials" OR XB (randomized controlled trial*))

AND

(XB (cancer) OR MH "Neoplasms" OR XB (oncology))

**Scopus**

(INDEXTERMS("attitude to health") OR INDEXTERMS("health knowledge, attitudes, practice") OR INDEXTERMS("patient preference") OR INDEXTERMS("choice behavior") OR

TITLE-ABS("patient* W/3 attitude*" OR "patient* W/3 preference*") OR TITLE("barrier*"))

AND

(INDEXTERMS("clinical trials as topic") OR INDEXTERMS("randomized controlled trials as topic") OR TITLE-ABS("clinical trial*" OR "randomized controlled trial*"))

AND

(INDEXTERMS("neoplasms") OR TITLE-ABS({cancer} OR {oncology}))

**Web of Science**

(TS=("attitude to health" OR "health knowledge, attitudes, practice" OR "patient preference" OR "choice behavior" OR

"patient* NEAR/3  attitude*" OR "patient* NEAR/3 preference*") OR TI=("barrier*"))

AND

(TS=("clinical trials as topic" OR "randomized controlled trials as topic" OR "clinical trial*" OR "randomized controlled trial*"))

AND

(TS=("neoplasms" OR "cancer" OR "oncology"))

**Appendix 2. Additional themes identified in the included studies**

Several reported barriers were identified in a small number of studies. Seven studies (5.3%) described fears related to increased discomfort, treatment burden or severity, or general procedural concerns as barriers to participation [1–7]. Privacy concerns were raised in 10 studies (7.6%) [8–17], including concerns about privacy breaches and that participation would lead to others finding out about their cancer. Cultural, spiritual, or religious barriers were described in 13 studies (9.9%) [3,4,10,12,18–26] ranging from broadly defined cultural influences, to a specific belief in prayer and faith over science, as reported by 27.3% of survey participants in Hillyer et al [3]. Markan et al. reported that the belief that God determines wellness and that illness and death are God’s will was higher amongst Black women compared to non-Black women [4]. Health- or age-related limitations were also cited by 18 studies [25,27–43], with 85.7% of those aged <65 and 72% aged >65 agreeing that their comorbidities impacted willingness to participate in trials [27]. In two studies, participants were hesitant to enroll due to fear of increased COVID-19 exposure [33,44]. Finally, four studies reported that previous denial of participation or previous negative experiences with trials were a barrier to future participation [29,45–47].

**References**

[1]	Brown RF, Cadet DL, Houlihan RH, et al. Perceptions of participation in a phase I, II, or III clinical trial among African American patients with cancer: what do refusers say? J Oncol Pract 2013;9:287–93. https://doi.org/10.1200/JOP.2013.001039.

[2]	Cohen MZ, Slomka J, Pentz RD, et al. Phase I participants’ views of quality of life and trial participation burdens. Support Care Cancer 2007;15:885–90. https://doi.org/10.1007/s00520-007-0216-0.

[3]	Hillyer GC, Beauchemin M, Hershman DL, et al. Discordant attitudes and beliefs about cancer clinical trial participation between physicians, research staff, and cancer patients. Clin Trials 2020;17:184–94. https://doi.org/10.1177/1740774520901514.

[4]	Markan U, Baker K, Eggleston C, et al. Psychosocial Factors That Influence a Woman’s Decision to Enroll in a Clinical Trial: Implications on How to Improve Clinical Trial Enrollment Among Black Women. Int J Radiat Oncol Biol Phys 2024;119:1347–56. https://doi.org/10.1016/j.ijrobp.2024.02.017.

[5]	Read K, Fernandez CV, Gao J, et al. Decision-making by adolescents and parents of children with cancer regarding health research participation. Pediatrics 2009;124:959–65. https://doi.org/10.1542/peds.2008-2878.

[6]	Sabesan S, Burgher B, Buettner P, et al. Attitudes, knowledge and barriers to participation in cancer clinical trials among rural and remote patients. Asia-Pac J Clncl Oncology 2011;7:27–33. https://doi.org/10.1111/j.1743-7563.2010.01342.x.

[7]	Coyne CA, Demian-Popescu C, Brown P. Rural cancer patients’ perspectives on clinical trials: a qualitative study. J Cancer Educ 2004;19:165–9. https://doi.org/10.1207/s15430154jce1903_11.

[8]	Avis NE, Smith KW, Link CL, et al. Factors associated with participation in breast cancer treatment clinical trials. J Clin Oncol 2006;24:1860–7. https://doi.org/10.1200/JCO.2005.03.8976.

[9]	Byrne MM, Tannenbaum SL, Glück S, et al. Participation in cancer clinical trials: why are patients not participating? Med Decis Making 2014;34:116–26. https://doi.org/10.1177/0272989X13497264.

[10]	Fairley R, Lillard JW, Berk A, et al. Increasing Clinical Trial Participation of Black Women Diagnosed with Breast Cancer. J Racial Ethn Health Disparities 2024;11:1701–17. https://doi.org/10.1007/s40615-023-01644-z.

[11]	Kong Q, Mei H, Lai Y, et al. Barriers and facilitators to participation in clinical trial among lymphoma patients from Sun Yat-sen University Cancer Center in China. Medicine (Baltimore) 2017;96:e8062. https://doi.org/10.1097/MD.0000000000008062.

[12]	Alotaibi M, Alsolami L, AlHarbi S, et al. Clinical Trial Awareness, Perceptions, and Participation Among Cancer Patients in Saudi Arabia. Healthcare (Basel) 2025;13:1044. https://doi.org/10.3390/healthcare13091044.

[13]	Brehaut JC, Carroll K, Gordon J, et al. Results from a Theory-Guided Survey to Support Breast Cancer Trial Participation: Barriers, Enablers, and What to Do about them. Current Oncology 2021;28:2014–28. https://doi.org/10.3390/curroncol28030187.

[14]	Dias AL, Chao JH, Lee D, et al. Patient perceptions concerning clinical trials in oncology patients. Contemp Clin Trials Commun 2016;4:179–85. https://doi.org/10.1016/j.conctc.2016.09.005.

[15]	Kubilay Tolunay P, Erol C, Kahraman S, et al. Understanding of Clinical Trials Among Patients With Cancer and Their Relatives. JAMA Netw Open 2025;8:e2457020. https://doi.org/10.1001/jamanetworkopen.2024.57020.

[16]	McKinney M, Bell R, Samborski C, et al. Clinical Trial Participation: A Pilot Study of Patient-Identified Barriers. Clin J Oncol Nurs 2021;25:647–54. https://doi.org/10.1188/21.CJON.647-654.

[17]	Rivera-Díaz M, García-Romero AN, Ayala-Marín AM, et al. Knowledge, Motivations and Concerns about Participation in Breast Cancer Clinical Trials in Puerto Rico. J Health Dispar Res Pract 2020;13:50–66.

[18]	Penberthy L, Brown R, Wilson-Genderson M, et al. Barriers to therapeutic clinical trials enrollment: Differences between African-American and White cancer patients identified at the time of eligibility assessment. Clin Trials 2012;9:788–97. https://doi.org/10.1177/1740774512458992.

[19]	Haynes-Maslow L, Godley P, Dimartino L, et al. African American women’s perceptions of cancer clinical trials. Cancer Med 2014;3:1430–9. https://doi.org/10.1002/cam4.284.

[20]	Tu S-P, Chen H, Chen A, et al. Clinical Trials: Understanding and Perceptions of Female Chinese-American Cancer Patients. Cancer 2005;104:2999–3005. https://doi.org/10.1002/cncr.21524.

[21]	Alhajji M, Bass SB, Nicholson A, et al. Comparing Perceptions and Decisional Conflict Towards Participation in Cancer Clinical Trials Among African American Patients Who Have and Have Not Participated. J Cancer Educ 2022;37:395–404. https://doi.org/10.1007/s13187-020-01827-w.

[22]	Chalela P, Suarez L, Muñoz E, et al. Promoting Factors and Barriers to Participation in Early Phase Clinical Trials: Patients Perspectives. J Community Med Health Educ 2014;4:1000281. https://doi.org/10.4172/2161-0711.1000281.

[23]	Hernandez ND, Durant R, Lisovicz N, et al. African American Cancer Survivors’ Perspectives on Cancer Clinical Trial Participation in a Safety-Net Hospital: Considering the Role of the Social Determinants of Health. J Cancer Educ 2022;37:1589–97. https://doi.org/10.1007/s13187-021-01994-4.

[24]	Hoadley A, Fleisher L, Kenny C, et al. Exploring Racial Disparities in Awareness and Perceptions of Oncology Clinical Trials: Cross-Sectional Analysis of Baseline Data From the mychoice Study. JMIR Cancer 2024;10:e56048. https://doi.org/10.2196/56048.

[25]	Lee GE, Ow M, Lie D, et al. Barriers and facilitators for clinical trial participation among diverse Asian patients with breast cancer: a qualitative study. BMC Womens Health 2016;16:43. https://doi.org/10.1186/s12905-016-0319-1.

[26]	Zyumbileva P, Spanic T, Gillanders E, et al. Understanding the patient journey: Barriers, facilitators, and expectations in joining a chemotherapy de-escalation trial among premenopausal patients with breast cancer. Breast 2026;87:104770. https://doi.org/10.1016/j.breast.2026.104770.

[27]	Ayodele O, Akhtar M, Konenko A, et al. Comparing attitudes of younger and older patients towards cancer clinical trials. J Geriatr Oncol 2016;7:162–8. https://doi.org/10.1016/j.jgo.2016.03.005.

[28]	Shnorhavorian M, Doody DR, Chen VW, et al. Knowledge of Clinical Trial Availability and Reasons for Nonparticipation Among Adolescent and Young Adult Cancer Patients: A Population-based Study. Am J Clin Oncol 2018;41:581–7. https://doi.org/10.1097/COC.0000000000000327.

[29]	Almutairi KM, Alonazi WB, Alodhayani AA, et al. Barriers to Cancer Clinical Trial Participation Among Saudi Nationals: A Cross-Sectional Study. J Relig Health 2017;56:623–34. https://doi.org/10.1007/s10943-016-0306-8.

[30]	Anderson C, Smitherman AB, Meernik C, et al. Patient/Provider Discussions About Clinical Trial Participation and Reasons for Nonparticipation Among Adolescent and Young Adult Women with Cancer. J Adolesc Young Adult Oncol 2020;9:41–6. https://doi.org/10.1089/jayao.2019.0078.

[31]	Arana-Chicas E, Prisco LMH, Sharma S, et al. Barriers to participation in clinical trials of rural older adult cancer survivors: A qualitative study. The Journal of Rural Health 2025;41:e12852. https://doi.org/10.1111/jrh.12852.

[32]	Barakat LP, Schwartz LA, Reilly A, et al. A Qualitative Study of Phase III Cancer Clinical Trial Enrollment Decision-Making: Perspectives from Adolescents, Young Adults, Caregivers, and Providers. J Adolesc Young Adult Oncol 2014;3:3–11. https://doi.org/10.1089/jayao.2013.0011.

[33]	Fleury ME, Farner AM, Unger JM. Association of the COVID-19 Outbreak With Patient Willingness to Enroll in Cancer Clinical Trials. JAMA Oncol 2021;7:131–2. https://doi.org/10.1001/jamaoncol.2020.5748.

[34]	Gansler T, Jin M, Bauer J, et al. Outcomes of a cancer clinical trial matching service. J Cancer Educ 2012;27:11–20. https://doi.org/10.1007/s13187-011-0296-x.

[35]	Go RS, Frisby KA, Lee JA, et al. Clinical trial accrual among new cancer patients at a community-based cancer center. Cancer 2006;106:426–33. https://doi.org/10.1002/cncr.21597.

[36]	Harrison JD, Solomon MJ, Young JM, et al. Surgical and oncology trials for rectal cancer: who will participate? Surgery 2007;142:94–101. https://doi.org/10.1016/j.surg.2007.01.013.

[37]	Kearns E, Chu AK, Nissim R, et al. The Supportive Care Needs of Individuals Living With Advanced or Metastatic Lung Cancer Receiving Targeted or Immunotherapies. Psycho-Oncology 2024;33:e70015. https://doi.org/10.1002/pon.70015.

[38]	Lu W, Li J, Gu S, et al. Attitude of patients with liver cancer toward clinical drug trials: empirical qualitative research. Sci Rep 2025;15:21026. https://doi.org/10.1038/s41598-024-83162-4.

[39]	Mandane B, Amirthanayagam A, Patel N, et al. Attitudes and barriers to participation in window-of-opportunity trials reported by White and Asian/Asian British ethnicity patients who have undergone treatment for endometrial cancer. Trials 2023;24:754. https://doi.org/10.1186/s13063-023-07572-x.

[40]	Petitte T, Nichols A, Narsavage G. Cultural and Environmental Enablers and Barriers to Participation and Retention in Clinical Trials for Cancer Research. EC Pulmonol Respir Med 2019;8:600–6.

[41]	Sutherland HJ, Lockwood GA, Till JE. Are we getting informed consent from patients with cancer? J R Soc Med 1990;83:439–43. https://doi.org/10.1177/014107689008300710.

[42]	Townsley CA, Chan KK, Pond GR, et al. Understanding the attitudes of the elderly towards enrolment into cancer clinical trials. BMC Cancer 2006;6:34. https://doi.org/10.1186/1471-2407-6-34.

[43]	Williams N, Lee YC, Russell H, et al. Listening to Australians with ovarian cancer: a cross-sectional survey investigating clinical trials awareness, information access and participation. Support Care Cancer 2026;34:350. https://doi.org/10.1007/s00520-026-10586-1.

[44]	Smith KL, Mead-Harvey C, Mazza GL, et al. TBCRC 057: Survey about willingness to participate in cancer clinical trials during the pandemic. Cancer Med 2024;13:e7090. https://doi.org/10.1002/cam4.7090.

[45]	Virani S, Burke L, Remick SC, et al. Barriers to Recruitment of Rural Patients in Cancer Clinical Trials. J Oncol Pract 2011;7:172–7. https://doi.org/10.1200/JOP.2010.000158.

[46]	Madsen SM, Mirza MR, Holm S, et al. Attitudes towards clinical research amongst participants and nonparticipants. J Intern Med 2002;251:156–68. https://doi.org/10.1046/j.1365-2796.2002.00949.x.

[47]	Kessel KA, Vogel MME, Kessel C, et al. Cancer clinical trials - Survey evaluating patient participation and acceptance in a university-based Comprehensive Cancer Center (CCC). Clin Transl Radiat Oncol 2018;13:44–9. https://doi.org/10.1016/j.ctro.2018.10.001.
